# Recent Progress in Nanotechnology Improving the Therapeutic Potential of Polyphenols for Cancer

**DOI:** 10.3390/nu15143136

**Published:** 2023-07-13

**Authors:** Italo Rennan Sousa Vieira, Leticia Tessaro, Alan Kelbis Oliveira Lima, Isabela Portella Silva Velloso, Carlos Adam Conte-Junior

**Affiliations:** 1 Analytical and Molecular Laboratorial Center (CLAn), Institute of Chemistry (IQ), Federal University of Rio de Janeiro (UFRJ), Cidade Universitária, Rio de Janeiro 21941-909, RJ, Brazil; 2 Center for Food Analysis (NAL), Technological Development Support Laboratory (LADETEC), Federal University of Rio de Janeiro (UFRJ), Cidade Universitária, Rio de Janeiro 21941-598, RJ, Brazil; 3 Laboratory of Advanced Analysis in Biochemistry and Molecular Biology (LAABBM), Department of Bio-Chemistry, Federal University of Rio de Janeiro (UFRJ), Cidade Universitária, Rio de Janeiro 21941-909, RJ, Brazil; 4 Graduate Program in Chemistry (PGQu), Institute of Chemistry (IQ), Federal University of Rio de Janeiro (UFRJ), Cidade Universitária, Rio de Janeiro 21941-909, RJ, Brazil; 5 Graduate Program in Food Science (PPGCAL), Institute of Chemistry (IQ), Federal University of Rio de Janeiro (UFRJ), Cidade Universitária, Rio de Janeiro 21941-909, RJ, Brazil; 6 Nanobiotechnology Laboratory, Institute of Biology (IB), Department of Genetics and Morphology, University of Brasilia, Brasilia 70910-900, DF, Brazil

**Keywords:** bioactive compounds, carcinogenesis, encapsulation, nanocarriers, nanomaterials, resveratrol, tumor

## Abstract

Polyphenols derived from fruits, vegetables, and plants are bioactive compounds potentially beneficial to human health. Notably, compounds such as quercetin, curcumin, epigallocatechin-3-gallate (EGCG), and resveratrol have been highlighted as antiproliferative agents for cancer. Due to their low solubility and limited bioavailability, some alternative nanotechnologies have been applied to encapsulate these compounds, aiming to improve their efficacy against cancer. In this comprehensive review, we evaluate the main nanotechnology approaches to improve the therapeutic potential of polyphenols against cancer using in vitro studies and in vivo preclinical models, highlighting recent advancements in the field. It was found that polymeric nanomaterials, lipid-based nanomaterials, inorganic nanomaterials, and carbon-based nanomaterials are the most used classes of nanocarriers for encapsulating polyphenols. These delivery systems exhibit enhanced antitumor activity and pro-apoptotic effects, particularly against breast, lung, prostate, cervical, and colorectal cancer cells, surpassing the performance of free bioactive compounds. Preclinical trials in xenograft animal models have revealed decreased tumor growth after treatment with polyphenol-loaded delivery systems. Moreover, the interaction of polyphenol co-delivery systems and polyphenol–drug delivery systems is a promising approach to increase anticancer activity and decrease chemotherapy side effects. These innovative approaches hold significant implications for the advancement of clinical cancer research.

## 1. Introduction

Cancer is one of the leading causes of mortality worldwide. According to data from the World Health Organization (WHO) [1], over 10 million cases were registered globally by 2020, and it is estimated that this number will rise to 19 million by 2040, primarily due to aging and population growth. While current cancer treatments, such as chemotherapy, radiotherapy, and surgery, have successfully treated various stages of cancer, WHO recommends adopting a healthy eating pattern to decrease risk factors and cancer incidence [2,3]. This includes consuming a diet rich in fruits, vegetables, and whole grains while reducing alcohol and tobacco consumption and maintaining regular physical activity [4]. Furthermore, numerous epidemiological studies have confirmed that a diet rich in fruits, vegetables, and grains can lower the risk of developing cancer [5,6]. These foods are rich sources of bioactive compounds, especially polyphenols and phenolic compounds, which have several benefits to human health due to their antioxidant, anti-inflammatory, antimicrobial, antimutagenic, and anticancer properties [7,8]. Moreover, polyphenols have been extensively researched for their preventive effects on various conditions, such as diabetes, obesity, cardiovascular disease, and neurodegenerative disorders [9,10].

**Figure 1:**
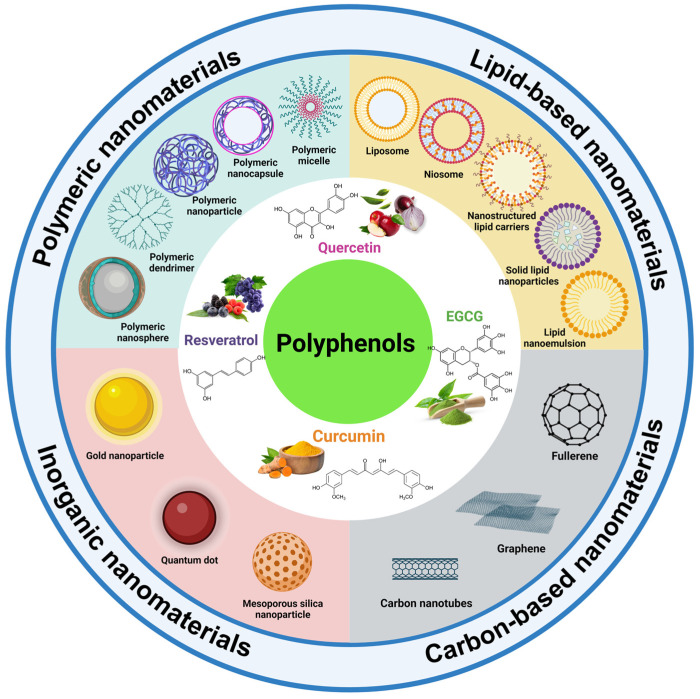
Main polyphenols with therapeutic potential against cancer and nanomaterials typically used as nanocarriers.

Among the polyphenolic compounds with therapeutic potential, quercetin, curcumin, epigallocatechin-3-gallate (EGCG), and resveratrol have gained a greater prominence in recent years, mainly due to their antioxidant properties, along with pro-oxidant, antiproliferative, anti-angiogenic, and anti-metastatic effects [11,12,13,14,15,16,17]. Despite the high curative potential, a low solubility and poor bioavailability hinder the proper gastrointestinal (GI) absorption of these phytonutrients [7]. To overcome these challenges, several approaches in nanotechnology have been proposed in recent years to improve the effectiveness of polyphenols, such as nanoencapsulation in delivery systems (Figure 1) [18,19,20,21]. These materials mainly perform protective functions against the hostile environment of the digestive system, assisting in a controlled and targeted delivery to the target tissue [19]. Lately, polyphenol-loaded delivery systems have shown promising results by interfering with specific stages of the carcinogenic process, inhibiting cell proliferation, and inducing apoptosis in various cancer cells [15].

Several drug delivery systems have been proposed in recent decades as an alternative to the nanoencapsulation of polyphenols applied in cancer therapy [22,23]. The main classes of nanocarriers include (i) polymeric nanomaterials (nanoparticles, nanocapsules, nanospheres, micelles, and dendrimers); (ii) lipid-based nanomaterials (liposomes, niosomes, nanostructured lipid carriers, solid lipid nanoparticles, and lipid nanoemulsions); (iii) inorganic nanomaterials (gold nanoparticles, quantum dots, and mesoporous silica nanoparticles); and (iv) carbon-based nanomaterials (carbon nanotubes, graphene and its derivatives, and fullerene) (Figure 1) [24,25,26,27]. These nanomaterials have been used in several in vitro studies and animal preclinical tests to elucidate their anticancer in vivo effects [28,29,30].

**Figure 2:**
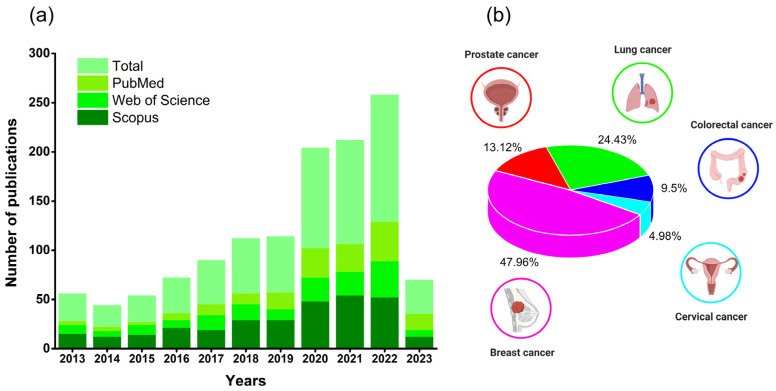
An overview of journal articles concentrating on the applications of polyphenol-loaded delivery systems in cancer therapies from 2013 to 2023 (25 April 2023) in relevance to (**a**) the number of publications and (**b**) types of cancer. The search was conducted in the Scopus, Web of Science, and PubMed databases using the keywords “nano-delivery system” OR “drug delivery systems” OR nanocarrier* OR nanoformulation* AND polyphenol* AND cancer* OR tumor*.

A review of the scientific literature using the Scopus, Web of Science, and PubMed databases (25 April 2023) showed that from 2013 to 2023, there was an increasing number of publications on polyphenol delivery systems for cancer therapy (Figure 2). Among the main types, breast (47.96%), lung (24.43%), and prostate (13.12%) cancers are the most frequently studied due to the more significant number of cases in individuals worldwide. For example, breast cancer was identified as the most prevalent type in the world, affecting about 2.3 million women by 2020, and causing the death of 685,000 individuals worldwide [1]. Thus, novel treatments based on nanoencapsulated natural products emerge as a potential alternative for cancer therapy, demonstrating the importance of this field of study.

Although some literature reviews address the potential of polyphenols for cancer therapy, most of these studies are focused on a mechanistic approach to active compounds at the cellular level and their potential effects on tumor tissue [8,31,32]. Thus, the objective of the current review was to address the therapeutic effect of nanocarried polyphenols from a series of delivery systems with potential for cancer therapy by evaluating in vitro studies and preclinical trials in animals through an overview of recent progress. Furthermore, this study addresses the use of polyphenol co-delivery systems or the combined therapy of polyphenol–drug delivery systems to decrease the side effects of anticancer drugs. Finally, this review concludes with future trends in polyphenol-loaded delivery systems for cancer and their outlook for clinical research.

## 2. Overview of Polyphenols and Their Anticancer Properties

**Figure 3:**
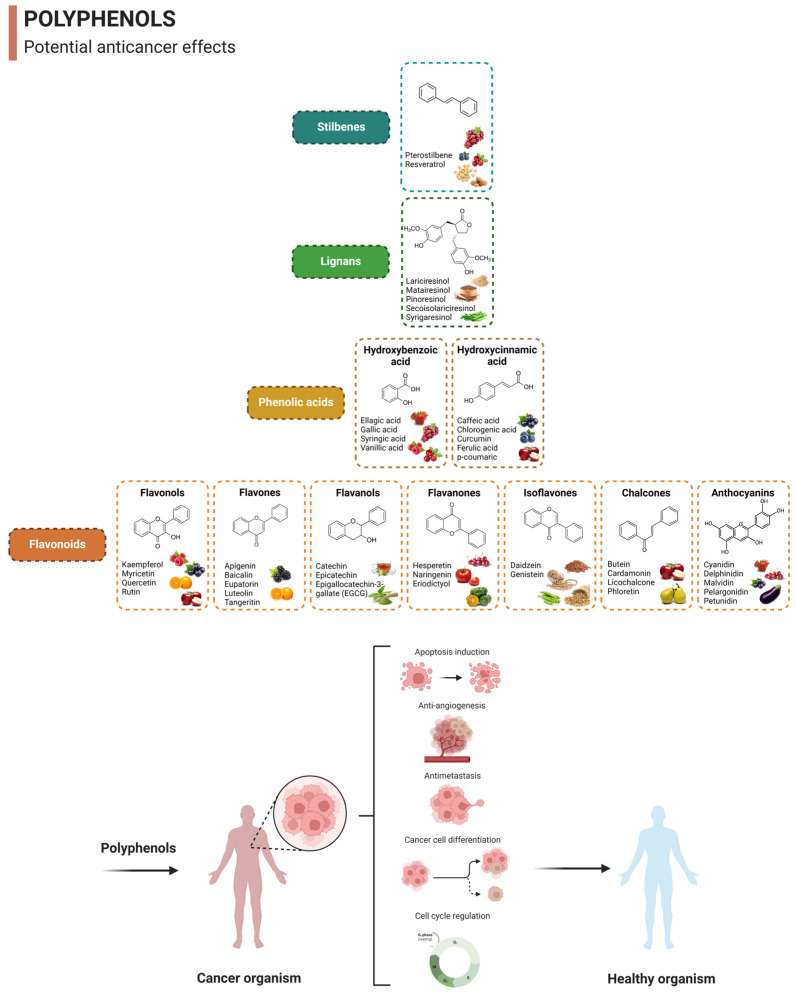
The main classes of polyphenols (stilbenes, lignans, phenolic acids, and flavonoids) and respective sub-classes are represented by the basic chemical structure of the compounds and their food sources. Their main anticancer effects include the induction of apoptosis, antiangiogenic and antimetastatic effects, and acting on cancer cell differentiation and cell cycle regulation.

Food-derived polyphenols are secondary metabolites widely found in fruits and vegetables and have several benefits to human health [7]. These aromatic compounds can be categorized into four main classes: (i) phenolic acids, (ii) flavonoids, (iii) stilbenes, and (iv) lignans [33,34]. Each sub-class has different chemical structures and biological properties (Figure 3). Lately, it has been proven that polyphenols exhibit excellent antioxidant and anti-inflammatory properties, acting as promising herbal medicines for the prevention and treatment of cancer [7]. Polyphenols can exert several anticancer effects, mainly by regulating the mechanism of cell death induced by apoptosis, a reduction in cell cycle arrest to inhibit cell proliferation, and antiangiogenic and antimetastatic potential from delimiting tumor growth, invasion, and autophagy (Figure 3) [8,18].

Table 1 shows some widely investigated polyphenols in cancer prevention and therapy. The main class, the sub-class, the food source, the type of cancer, and the main therapeutic effects on cancer cells are also presented. In the following topics, some polyphenols and the main approaches in nanotechnology to improve the therapeutic potential of these bioactive compounds in cancer therapy are presented through reviewed studies.

### 2.1. Quercetin

Quercetin (3,3’,4’,5,7-pentahydroxyflavone) is a polyphenol of the flavonoid class and sub-class of flavonols, widely found in citrus fruits, blackberries, cranberries, blueberries, and green vegetables [11,15]. Its antioxidant properties are well-proven and established in the scientific literature as a hallmark of its anticancer activity. Previous studies have shown that quercetin can act on tumor cells by inducing apoptosis, inhibiting protein kinase C, modulating the cell cycle, and inhibiting angiogenesis [15]. Furthermore, it has recently been reported that quercetin has synergistic effects when combined with traditional chemotherapy [11].

Recently, Hashemzaei et al. (2017) [15] evaluated the effect of quercetin in the treatment of breast and prostate cancers from preclinical studies in mice. The treatment consisted of the application of different concentrations of quercetin (50, 100, and 200 mg/kg) in mice and 5% dextrose as a control group. The authors observed significant differences in breast tumor sizes after 18 days of treatment and also in survival rates between a treated group (200 mg/kg) and the control. However, no significant difference was observed in the survival rate of the quercetin-treated group at 50 mg/kg and the control group. For prostate cancer, a great inhibition of tumor cell growth and the induction of apoptosis was observed [15].

Cell viability for cervical and lung cancers in the presence of quercetin was also verified. The action of quercetin on cervical cancer cells promoted cell cycle arrest in the G2/M phase and cell apoptosis, also inhibiting cell migration and invasion [36]. The A-549 cell line was used for lung cancer in in vitro studies. An apoptosis rate of 12.96 and 24.58%, respectively, was observed when the cells were incubated with 1.2 μmol/L of quercetin for 48 and 72 h. The results also show the inhibition of A-549 cell growth. These facts may be associated with a reduced expression of the apoptosis regulatory gene Bcl-2 and increased expression of the apoptosis regulator gene Bax [35].

Several studies describe quercetin as a unique compound for its high potential to fight cancer-related diseases in many ways. Different concentrations have been evaluated, proving that this bioactive compound suppresses tumor growth of various cancer cell lines, including breast, colorectal, cervical, and lung, among others [43].

### 2.2. Curcumin

Curcumin is the most important component of the rhizomes of *Curcuma longa* L. (turmeric) and has a great bioactive potential with antitumor, antioxidant, and anti-inflammatory properties [37]. The anticancer activity of this compound is associated with the mechanism of action that induces apoptosis and inhibits tumor proliferation and invasion by suppressing a variety of cell signaling pathways. Curcumin also inhibits the signaling of the STAT3 and NF-ĸB pathways that play an important role in cancer development [12]. The literature brings together several studies using curcumin for different types of cancer, which are presented in Table 1, evidencing its ability to act on several cancer cell lines.

In a study by Kazemi-Lomedasht et al. (2013) [37], curcumin showed anticancer effects against T47D breast cancer cells. The extract of curcumin prepared at the concentration of 22 μM increased the inhibition of the telomerase gene expression in the T47D cell line and decreased cell viability by 50% in 48 h [37]. On the other hand, testing for the cervical cancer cell line HeLa at a concentration of 34.23 μM/mL reduced cell proliferation and inhibited NF-kB and Wnt signaling pathways, resulting in cell death [16]. The turmeric extract also showed prostate cancer cell uptake and a decreased cell viability in about 60% of the DU145 and C4-2 cells when applied at 18.5 μM and 19.6 μM concentrations, respectively [38].

Finally, the turmeric extract was evaluated in models of a colorectal cancer xenograft in mice induced by AOM-DSS (azoxymethane–dextran sodium sulfate) [39]. The animals received 500 mg/kg per day using oral gavage for 7 weeks. The expression levels of IL-1β, IL-6, Cox-2, and β-catenin decreased significantly, demonstrating that the turmeric extract suppresses the tumorigenesis induced by AOM-DSS by downregulating cytokine expression. It was also found that the number of colorectal tumors and tumor size decreased significantly [39].

The data published in the literature show the potential of turmeric to promote safe and effective therapies against cancer, including in vitro and in vivo tests, which may assist in the treatment of novel therapeutic targets.

### 2.3. Epigallocatechin-3-Gallate (EGCG)

EGCG is a polyphenol with medicinal potential mainly found in green tea (*Camellia sinensis*) [44]. Among its properties include antioxidant, antimicrobial, anti-inflammatory, antifungal, anti-viral, anti-cardiovascular, and antihypertensive activity [14]. Antioxidant action is very important and crucial against some diseases, including cancer, as it can hinder carcinogenesis by targeting epigenetic changes [45].

Chen et al. (2020) [42] used the H1299 lung cancer cell line to evaluate its anticancer action. The results show that EGCG could suppress the proliferation of H1299 cells in concentrations greater than 20 μM. A reduction of about 60% of cell viability in 72 h at the concentration of 36.03 μM was found. The colony-forming activity of H1299 cells was completely suppressed at the concentration of 5 μM [42]. Similarly, the same concentration of EGCG (20 μM) was evaluated in another study for MCF-7 and MDA-MB-231 breast cancer cells [40]. After 24 h of incubation, cells treated with EGCG showed a significant increase in E-cadherin and suppression of vimentin expression in both mRNA and protein levels. Furthermore, a decrease in DNA methyltransferase (DNMT) expression and activity was observed in breast cancer cells [40].

In a study by Chen et al. (2019) [41], the EGCG was lipophilized with ethanol and 5% Lipozyme TLIM, forming the LEGCG derivate. The effects of LEGCG on the proliferation of DU145 prostate cancer cells were evaluated. The treatment with LEGCG at concentrations of 0, 10, 20, and 40 μg/mL for 24 h showed DU145 cell apoptosis rates of 8.63%, 12.78%, 25.62%, and 58.51%, respectively. Compared to a study by Ravindranath et al. (2006) [13], the used EGCG at 10, 20, 40, and 80 μg/mL for 48 h led to 13.9%, 19.1%, 42.2%, and 58.1% apoptosis in DU145 cells. These studies prove the antiproliferation capacity using EGCG and its LEGCG derivate for the DU145 cell line in 48 h at a 50 μg/mL concentration per MTT assay.

### 2.4. Resveratrol

Resveratrol occurs naturally in many plants but is also found in foods included in the diet, such as grapes, berries, and peanuts [14]. This bioactive compound has an antioxidant action resulting in several health benefits. Furthermore, research shows that resveratrol also has antimicrobial and antifungal activity [34] and can be used in the treatment of various diseases, including cancer [14,17].

Recently, Wang et al. (2022) [31] evaluated the effect of the resveratrol compound against HeLa cervical cancer cells. The results obtained at the concentration of 71 μM showed a significant decrease in ESR1, AKT1, and MAPK3 expression. Moreover, resveratrol suppressed migration and inhibited the phosphorylation of ERK1/2, consequently inhibiting the growth of HeLa cells [31].

The anticancer potential of resveratrol was also investigated against lung cancer lines A549 and H1299 in a study by Liang et al. (2023) [17]. The authors observed a remarkable decrease in cell viability for A549 and H1299 cells at concentrations of 50 μM and 25 μM, respectively, after 24 h. However, in 48 h, cell viability was significantly inhibited by about 50% at 25 μM for H1299 and A549 cells. Morphological changes were observed through the appearance of a heterogeneous population in the cells, and compared with the control group, they were flattened and elongated, which occurs in senescent cells. Furthermore, inhibiting tumor growth induced by cell senescence at 25 μM led to cell apoptosis. The authors’ proposed mechanism is that cellular inhibition occurs in the G1 phase, accompanied by changes in apoptotic proteins (Bax, Bcl-2, and cleaved caspase-3). This suggests that resveratrol causes a disturbance in lung cancer cellular homeostasis by destroying the pool of intracellular antioxidants that are often active in cancer to increase the ROS production that regulates the expression of CAT, HO-1, NQO1, and SOD1 proteins. These findings demonstrate novel perspectives on mechanisms of action of resveratrol in lung cancer [17].

## 3. Application of Nanotechnology Improving the Efficiency of Polyphenols for Cancer

Nanotechnology plays a crucial role in the therapeutic efficacy of novel natural compounds in cancer treatments. As reported before, polyphenols derived from food sources exhibit significant potential in cancer prevention. However, they encounter challenges primarily related to water solubility, stability, and limited bioavailability. As a result, several nanotechnology-based approaches have been proposed to enhance the therapeutic effect of polyphenols for cancer treatment in recent years. Furthermore, the combination of polyphenols and drugs in delivery systems appears as a strategy to reduce the side effects of anticancer drugs in conventional therapy.

Table 2 shows some current studies on applying nanomaterials as potential nanocarriers of polyphenolic compounds used in cancer therapy.

### 3.1. Polyphenol-Loaded Delivery Systems

#### 3.1.1. Polymeric Nanomaterials

Polymeric nanomaterials have been widely applied as nanocarriers of drugs and biomolecules for cancer therapy, primarily due to their unique properties, such as an adjustable size, shape, surface chemistry, and biocompatibility [46,47]. These materials can be designed to encapsulate and release therapeutic agents into tumor cells, mainly as polymer nanoparticles, nanocapsules, nanospheres, polymer micelles, and dendrimers, resulting in better treatment outcomes [7]. Some polymeric materials applied in cancer therapy include biodegradable polymers such as poly(lactide-co-glycolide) (PLGA), poly(lactic acid) (PLA), poly(caprolactone) (PCL), poly(ethylene glycol) (PEG), and their copolymers [48,49], in addition to polysaccharide-based biopolymers, such as chitosan, sodium alginate, hyaluronic acid, and proteins (such as albumin, gelatin, casein, and elastin) [50,51]. These nanomaterials can still be functionalized for a specific targeted delivery and have been extensively investigated for chemotherapy, immunotherapy, and gene therapy applications [52].

- Polymeric nanoparticles

Polymeric nanoparticles are small particles made of synthetic or natural polymers with sizes typically ranging from 1 to 1000 nm [49]. They offer numerous advantages for drug delivery and are extensively investigated for their applications in releasing bioactive compounds for cancer treatment [46]. Several types of polymeric nanoparticles are commonly used: polymeric nanocapsules, polymeric nanospheres, polymeric micelles, dendrimers, and cyclodextrin, among others [7].

Recently, Mahdian et al. (2023) [24] evaluated the anticancer potential of curcumin loaded into chitosan nanocapsules within gelatin hydrogel as a stimulus-responsive drug delivery system. A decrease in cell viability against CT26 colon cancer cells (IC_50_ = 24.16 μg/mL) was observed after 72 days of treatment. The percentage of CT26 cells that underwent cell death increased with time. The authors suggested that this effect may be associated with an increase in the pH of the cellular environment, which becomes more acidic over time, and provides a greater drug release.

**Table 2:** Polyphenol nanocarriers: In vitro studies using different cancer cell lines.

Polyphenol	Nanocarrier/ Nanoformulation	Particle Size	Cell Lines	Cell Viability Studies		Publication Year	References
				Free Compound	Nanocarrier		
Quercetin	Chitosan/clay/graphitic-carbon nitride nanocomposite hydrogel	454.65 nm	MCF-7 human breast cancer cell line	Cell viability = 100% (to 100 µM)	Cell viability = 60% (to 100 µM)	2023	[19]
	Chitosan/SBE-β-CD nanoparticles	272.07 nm	HeLa cervical cancer cells	IC_50_ = 59.84 µM Cell viability = 36.24% (to 150 µM)	IC_50_ = 66.68 at 43.55 µM Cell viability = 20.12 at 6.94% (to 150 µM)	2023	[28]
	Copper nanocluster-doped hydroxyapatite nanoparticles	36.2 nm	HeLa cervical cancer cells	IC_50_ = 300 µM Cell viability = 70% (to 500 µM)	IC_50_ = 200 µM Cell viability = 30% (to 500 µM)	2021	[29]
	Gelatin/PVP/GO nanocarrier	468 nm	MCF-7 human breast cancer cell line	Cell viability = 51.15%	Cell viability = 46.85%	2023	[20]
	GO nanocomposite film	-	BT474 breast cancer cells	IC_50_ = ND Cell viability = ~100%	IC_50_ = 99.29 µg/mL Cell viability = ~15%	2022	[53]
	Nanocochleates	502 nm	5000 KB human mouth cancer cells	IC_50_ = 5 µg/mL Cell viability = 20% (to 40 µg/mL)	IC_50_ = 10 µg/mL Cell viability = 40% (to 40 µg/mL)	2022	[30]
	PVP/PVA/TiO_2_ nanocomposite hydrogel	330 nm	U87 human glioblastoma cell line	Cell viability = 61%	Cell viability = 74%	2023	[54]
	Vitamin-E TPGS nanoemulsion	200 nm	HCT-116 colon cancer cell line	Cell viability = ~0 38% (to 100 µM)	Cell viability = ~0 25% (to 100 µM)	2022	[55]
			HT-29 colon cancer cell line	Cell viability = ~50% (to 100 µM)	Cell viability = ~0 35% (to 100 µM)		
Curcumin	Black-seed-oil-based nanoemulsion	28.53 nm	MCF-7 human breast cancer cell line	IC_50_ = 6.67 µg/mL Cell viability = ~50% (to 6 µg/mL)	IC_50_ = 4.76 µg/mL Cell viability = ~35% (to 6 µg/mL)	2020	[56]
	Chitosan-based microspheres	5 nm	MDA-MB 231 model breast cancer cells	Cell viability = ~45% (to 96 µM)	Cell viability = ~50% (to 96 µM)	2020	[57]
	Curcumin–lactoferrin conjugated nanostructures	166 nm	HCT116 human colon cancer cells	IC_50_ = 3.3 µg/mL Cell viability = 15% (to 5.2 µg/mL)	IC_50_ = 0.5 µg/mL Cell viability = 10% (to 5.2 µg/mL)	2018	[58]
	Emulsome nanoparticles	184.21 nm	LNCaP prostate cancer cell line	IC_50_ = ND Cell viability = ~75% (to 30 µM)	IC_50_ = 17.1 µM Cell viability = 66% (to 30 µM)	2023	[59]
	Fe_3_O_4_/chitosan/agarose nanoemulsion	279 nm	MCF-7 human breast cancer cell line	IC_50_ = ND Cell viability = ~60% (to 100 µM)	IC_50_ = 17.1 µM Cell viability = 48% (to 100 µM)	2023	[60]
	Fe_3_O_4_/PEG/folic acid nanoparticles	650.1 nm	MCF-7 human breast cancer cell line	IC_50_ = 49.91 µM Cell viability = <10% (to 100 µM)	IC_50_ = ND Cell viability = >35% (to 100 µM)	2022	[61]
			A549 lung cancer cell	IC_50_ = 50.75 µM Cell viability = <10% (to 100 µM)	IC_50_ = ND Cell viability = ~40% (to 100 µM)		
	PLGA/levan nano-micelles	154.16 nm	MCF-7 human breast cancer cell line	IC_50_ = 0.01323 mg/mL Cell viability = ~75% (to 0.006 mg/mL)	IC_50_ = 0.01120 mg/mL Cell viability = ~65% (to 0.006 mg/mL)	2021	[62]
	PGS-based nanoparticles	121 nm	HeLa cervical cancer cell	IC_50_ = 21.27 µM Cell viability = <25% (to 0.02 mg/mL)	IC_50_ = 15.95 µM Cell viability = <25% (to 0.02 mg/mL)	2022	[63]
	Pyromellitic dianhydride crosslinked cyclodextrin nanosponges	70 nm	MCF-7 human breast cancer cell line	Cell viability = ~50% (to 130 mg/mL)	Cell viability = ~80% (to 200 mg/mL)	2019	[64]
EGCG	Gold nanoparticles	90.3 nm	A375SM human melanoma cell line	IC_50_ = ND Cell viability = ~60% (to 31.8 μM)	IC_50_ = 67.6 µg/mL Cell viability = ~20% (to 200 μM)	2019	[65]
			MDA-MB-231 human breast cancer cell line	IC_50_ = ND Cell viability = ~60% (to 31.8 μM)	IC_50_ = 54.7 µg/mL Cell viability = 0% (to 200 μM)		
			MIA PaCa-2 human pancreatic cancer cell line	IC_50_ = ND Cell viability = ~60% (to 31.8 μM)	IC_50_ = 17.0 µg/mL Cell viability = ~10% (to 200 μM)		
			PC3 human prostate cancer cell line	IC_50_ = ND Cell viability = ~40% (to 31.8 μM)	IC_50_ = 24.9 µg/mL Cell viability = ~5% (to 200 μM)		
	Lecithin and non-ionic surfactant nanoemulsion	10 nm	H1299 human lung cancer cell line	IC_50_ = 36.03 μM Cell viability = ~70% (to 40 μM)	IC_50_ = 4.71 μM Cell viability = ~50% (to 40 μM)	2020	[42]
			A549 human lung cancer cell line	IC_50_ = ND Cell viability = ~70% (to 40 μM)	IC_50_ = 16.05 μM Cell viability = ~65% (to 40 μM)		
	PLGA nanoparticles	175.8 nm	A549 lung cancer cell line	IC_50_ = 72.63 μM Cell viability = 85% (to 25 μM)	IC_50_ = 19.57 μM Cell viability = 35% (to 25 μM)	2020	[66]
			H1299 lung cancer cell line	IC_50_ = 68.73 μM Cell viability = 85% (to 25 μM)	IC_50_ = 16.98 μM Cell viability = 20% (to 25 μM)		
	Self-assembled PEG and chlorin e6 (Ce6) nanoparticles	190–132 nm	4T1 mouse breast carcinoma cell line	Cell viability = >20% (to 100 μg/mL)	Cell viability = <10% (to 100 μg/mL)	2020	[67]
			A549 human non-small-cell lung cancer cell line	Cell viability = >15% (to 100 μg/mL)	Cell viability = <10% (to 100 μg/mL)		
			HCT116 human colorectal cancer cell line	Cell viability = >15% (to 100 μg/mL)	Cell viability = <10% (to 100 μg/mL)		
	Surface-active maghemite nanoparticles (SAMNs)	208.4 nm	HeLa human cervical cancer cells	Cell viability = ~125% (to 50 μg/mL)	Cell viability = ~40% (to 50 μg/mL)	2021	[68]
Resveratrol	Folic acid/PNIPAM hydrogels	243.59 nm	MCF-7 human breast cancer cell line	IC_50_ = 26.27 μg/mL Cell viability = ~40% (to 100 μg/mL)	IC_50_ = 3.55 μg/mL Cell viability = ~30% (to 100 μg/mL)	2023	[69]
	Gold nanoparticles crosslinked with PVP	41 nm	PANC-1 human pancreatic cancer cell line	Cell viability = ~30% (to 40 μM)	Cell viability = ~20% (to 15 μM)	2022	[26]
	Mesoporous silica nanoparticles	60 nm	MNT-1 human melanoma cell line	IC_50_ = 37.9 μM Cell viability = ~40% (to 50 μM)	IC_50_ = 25.5 μg/mL Cell viability = ~20% (to 100 μg/mL)	2021	[70]
			A375 human melanoma cell line	IC_50_ = 0.0026 μM Cell viability = ~10% (to 50 μM)	IC_50_ = 29.5 μg/mL Cell viability = ~5% (to 100 μg/mL)		
	PLGA/chitosan nanoparticles	341.56 nm	H1299 human non-small cell lung carcinoma cell line	IC_50_ = 57.31 μg/mL Cell viability = ~30% (to 100 μg/mL)	IC_50_ = 34.99 μg/mL Cell viability = ~15% (to 100 μg/mL)	2020	[71]
	Pluronic F127/vitamin-E TPGS micelles	318 nm	MCF-7 human breast cancer cell line	ND	IC_50_ = 0.93 μg/mL Cell viability = ~20.7 (to 2.5 μg/mL)	2021	[72]
			MDA-MB-231 human breast cancer cell line	ND	IC_50_ = 0.76 μg/mL Cell viability = 7.1% (to 2.5 μg/mL)		
	SBE-β-CD nanoparticles	264.2 nm	A549 lung cancer cell line	IC_50_ = 50.79 μM Cell viability = >50% (to 50 μM)	IC_50_ = 3.31 μM Cell viability = <10% (to 50 μM)	2020	[73]
			H358 lung cancer cell line	IC_50_ = 49.96 μM Cell viability = >50% (to 50 μM)	IC_50_ = 0.97 μM Cell viability = <10% (to 50 μM)		
			H460 lung cancer cell line	IC_50_ = 32.67 μM Cell viability = >30% (to 50 μM)	IC_50_ = 4.04 μM Cell viability = <10% (to 50 μM)		
			H4006 lung cancer cell line	IC_50_ = 133.43 μM Cell viability = <70% (to 50 μM)	IC_50_ = 3.10 μM Cell viability = <10% (to 50 μM)		
			H157 lung cancer cell line	IC_50_ = 30.81 μM Cell viability = >30% (to 50 μM)	IC_50_ = 5.42 μM Cell viability = <10% (to 50 μM)		
	Starch and chitosan films	-	AGS human gastric epithelial cell lines	IC_50_ = 31.1 μg/mL	IC_50_ = 91.1 μg/mL	2022	[74]

Legend: EGCG = epigallocatechin-3-gallate; GO = graphene oxide; ND = not determined; PCL = poly(*ε*-caprolactone); PEG = poly(ethylene glycol); PGS = poly(glycerol sebacate); PLGA = poly(lactide-co-glycolide); PNIPAM = poly(N-isopropylacrylamide-maltodextrin); Pluronic F127 = an amphiphilic triblock copolymer composed of poly(ethylene oxide) (PEO) and poly(propylene oxide) (PPO) (PEO-x-PPO-y-PEO-x); PVA = poly(vinyl alcohol); PVP = poly(vinyl pyrrolidone); SBE-β-CD = sulfonyl-ether-β-cyclodextrin; Vitamin-E TPGS = composed of D-tocopheryl polyethylene glycol succinate; ZnO = zinc oxide.

In a study by Zhou et al. (2020) [75], quercetin loaded onto PLGA-TPGS nanoparticles was evaluated for treating triple-negative breast cancer (TNBC), which demonstrates highly aggressive tumor biology. Spherical nanoparticles (particle size: 198.4 nm) exhibited significantly improved TNBC cell growth and metastasis inhibition. Furthermore, a remarkable antitumor effect of quercetin-loaded nanoparticles in 4T1-breast-tumor-bearing mice was observed with a tumor inhibition rate of 67.88% and a decrease in metastatic pulmonary colonies. In another study, ellagic acid loaded onto chitosan nanoparticles coated with a non-ionic surfactant (Tween^®^ 80) showed a potential effect against breast cancer investigated from in vitro and in vivo assays [76]. The coating material improved the formulation’s effectiveness and showed more significant cytotoxicity against MCF-7 tumor cells and biocompatibility in normal HEK293T cells. In in vivo assays, the percentage of tumor growth in mice treated with ellagic-acid-loaded nanoparticles was higher (73%) than the free compound (48%) after 28 days of treatment. Thus, the in vivo permeation of the nanoparticulate drug can improve the bioavailability of a polyphenol, increasing the therapeutic effect for tumor treatment.

- Polymeric micelles

Polymeric micelles are self-assembled structures generally formed by amphiphilic block copolymers in aqueous solutions [77]. These micelles have a core-shell structure, where the hydrophobic blocks form the core, while the hydrophilic blocks form the shell [78]. Polymeric micelles have been widely applied to deliver drugs and bioactive compounds that exhibit anticancer activity [72]. In this case, the hydrophobic core of the micelles can encapsulate hydrophobic drugs, while the hydrophilic shell provides stability and prevents aggregation [79]. Thus, the encapsulation of hydrophobic drugs within the micelle core increases their aqueous solubility, allowing for a better drug delivery and bioavailability [78].

For example, curcumin loaded from sodium-alginate-based polymeric micelles exerted a potent in vitro cytotoxicity against MC38-CEA murine colon cancer cell lines [80]. The colloidally stable micelles (particle size: 200 nm) also showed in vivo antitumor activity against two mice tumor models: MC38-CEA colon carcinoma and 4T1 breast carcinoma. The results confirmed that polymeric micelles are a potential alternative for encapsulating and delivering hydrophobic drugs such as curcumin, improving in vivo bioavailability. Similarly, curcumin loaded into self-assembled polymeric micelles from an amphiphilic peptide and stearic acid promoted high tumor activity against HepG2 hepatoma cells [81]. Curcumin-loaded polymeric micelles (particle size: ~50 nm) showed much more significant cell growth inhibition in HepG2 cells and less cytotoxicity in normal liver-derived L02 cells than free curcumin. Self-assembled micelles loaded on curcumin more precisely and efficiently overexpressed HepG2 cells due to an integrin targeting sequence (RGD) and cell penetration peptide (Octaarginine—R8). Combined with the higher drug solubility, specific property of pH sensitivity, and good tumor targeting, self-assembled micelles may have the clinical potential for delivering tumor targeting.

- Polymeric dendrimers

Polymeric dendrimers are highly branched macromolecules composed of a core, inner layers, and outer functional groups [82]. Dendrimers can be synthesized with precise control over their size, shape, and surface properties [83]. By changing the surface functional groups, dendrimers can be designed to target cancer cells or specifically accumulate in tumor tissues [84]. Thus, these structures can encapsulate or attach bioactive compounds onto their surface, acting as carriers or delivery systems [85]. Furthermore, dendrimers can protect encapsulated drugs from degradation and enhance their solubility, stability, and controlled release [83].

Zeynalzadeh et al. (2023) [86] recently studied curcumin’s structural and anticancer properties loaded on a poly(amido amine) dendrimer. The dendrimer complex synthesized up to the fourth generation (G4) and a modification with glycidol (G4–OH) was prepared using folic acid as an active targeting ligand for cancer cells. The results suggest that surface modification improves the drug complex’s growth inhibition performance on both cell lines MG-63 (osteosarcoma cell line) and HT-29 (human colorectal adenocarcinoma cell line). These findings suggest that curcumin, in combination with a fourth-generation dendrimer, may be an effective anticancer agent. Similarly, Alfei et al. (2020) [87] developed a dendrimer nanodevice containing gallic acid as a novel strategy to combat chemoresistance in human neuroblastoma cells. The main findings show that the pro-oxidant action of gallic acid on dendrimer systems increased the production of ROS in neuroblastoma cells (HTLA-230 human stage-IV cells), even at low concentrations. This strategy may become helpful in vivo-wise to increase the sensitivity of tumors to antineoplastic agents, decreasing the cytotoxic doses of drugs and, therefore, their systemic toxicity.

#### 3.1.2. Lipid-Based Nanomaterials

A series of lipid-based nanomaterials are currently applied in treating cancer and other diseases [88]. The most densely employed technologies based on lipid nanomaterials for loading polyphenols are liposomes, niosomes, nanostructured lipid carriers (NLCs), solid lipid nanoparticles (SLNs), and lipid nanoemulsions [25,42,89,90,91]. These materials have been widely applied in cancer therapy, mainly due to their biocompatibility, which makes them well tolerated by the human body [92]. Furthermore, they can be modified to exhibit specific targeting properties, allowing for the selective delivery of drugs to cancer cells, thereby minimizing side effects in healthy tissues [93].

- Liposomes

Liposomes are microscopic vesicles composed of lipid bilayers, typically spherical in shape, and similar in structure to cell membranes [7]. Lately, liposomes have been studied for drug and bioactive molecule encapsulation as delivery systems for cancer therapy [30,94]. Nanosystems can encapsulate anticancer drugs within their lipid bilayers or aqueous core, thus offering several benefits, including protection against degradation, an increased stability, and an improved circulation time in the body [25,95].

Recent studies have demonstrated the potential of liposome formulations to increase the bioavailability of polyphenols for cancer. In a study by Hasan et al. (2020) [25], curcumin loaded into chitosan-coated liposomes derived from different lecithin sources (salmon, soy, and rapeseed) showed the tumor growth inhibition of MCF-7 breast cancer cells. The results showed that chitosan-coated liposomes from salmon lecithin had a better anticancer effect than free curcumin (at 5 µM), indicating that liposomes could improve the efficacy of chemotherapy. Gholami et al. (2023) [96] reported similar results in studying liposome formulations based on soybean phosphatidylcholine (SPC) and hydrogenated SPC (HSPC) to increase the bioavailability of curcumin in HTB9 bladder cancer cells. The main results showed that liposomal curcumin effectively inhibited cancer cell viability, inducing apoptosis and DNA damage. Thus, liposome nanoparticles can significantly increase the stability and bioavailability of curcumin, which are essential in improving its pharmacological effect.

- Niosomes

Like liposomes, polyphenol-loaded niosomes have been extensively studied for their potential use in cancer therapy [97]. Niosomes are vesicular systems mainly formed by an aqueous core surrounded by chains of non-ionic surfactants, cholesterol, or its derivatives [98]. These allow the encapsulation of drugs and hydrophilic or lipophilic compounds [7]. Several studies have investigated the use of polyphenol-based niosomes for cancer therapy. For example, curcumin-loaded niosomes with a calcium alginate shell have been shown to have anticancer effects against MDA-MB-231 and SKBR3 breast cancer cells [89]. The niosomes increased curcumin’s cellular uptake and bioavailability, improving anticancer activity. Similarly, niosome formulations coated with PEG loaded with green tea polyphenols have also been studied for their anticancer effects [99]. The niosomes had dose-dependent toxicity against three different MCF-7, HepG2, and HL-60 cancer cell lines. Furthermore, niosomes proved to be plasma-stable and pH-sensitive, improving the green tea extract’s bioavailability and cytotoxic effect.

In general, niosomes offer a promising platform for drug delivery in cancer therapy, with their ability to encapsulate hydrophilic and hydrophobic drugs and their potential for drug-targeted delivery [100]. Furthermore, niosomes could play an essential role in improving the efficacy and reducing the toxicity of chemotherapy [101].

- Nanostructured lipid carriers (NLCs)

NLCs are lipid-based nanoparticle systems that have gained attention for their potential use in cancer therapy. NLCs are composed of solid or liquid lipids formed by a core stabilized by a surfactant, which can encapsulate hydrophobic drugs [102]. NLCs have several advantages over other nanoparticle systems, including an improved drug loading capacity, enhanced drug stability, and controlled drug release [103]. Furthermore, they have shown a low toxicity and biocompatibility in preclinical studies [104].

In cancer therapy, polyphenol-loaded NLCs can deliver anticancer drugs to tumors with a high precision and efficacy [105,106]. They can also enhance the cellular uptake of drugs by cancer cells, leading to an improved drug bioavailability and therapeutic outcomes. NLCs have been used to deliver natural compounds with anticancer properties, such as curcumin and resveratrol [90,107].

In a study by Gadag et al. (2021) [90], resveratrol-loaded NLCs were developed for localized drug delivery to mice mammary tissues. Using microneedle arrays, resveratrol-loaded NLCs showed an increased permeation of resveratrol into the skin, improving its bioavailability over oral administration. Moreover, the anticancer activity of resveratrol was enhanced in MDA-MB-231 breast cancer cell lines when delivered through resveratrol-loaded NLCs, resulting in an improved internalization compared to pure resveratrol. Thus, resveratrol-loaded NLCs delivered by the microneedle array system are an effective strategy for the local delivery of resveratrol for breast cancer therapy. In another study, oleuropein, a natural polyphenol in olive tree leaves (*Olea europaea* L.), was encapsulated in NLCs to improve the antioxidant power in A549 lung cancer cell lines [108]. The authors observed that NLCs enhanced oleuropein’s antioxidant activity, increasing the phytochemical’s protective ability against oxidative stress in cancer cells. These findings suggest that using NLCs to transport the natural polyphenol to lung epithelial cells could be a viable alternative.

- Solid lipid nanoparticles (SLNs)

SLNs are colloidal systems composed of solid lipids, with a particle diameter typically ranging from 10 to 1000 nm [7]. SLNs have a core-shell structure, where a stabilizing shell surrounds the solid lipid core, normally formed by a lipid matrix of triglycerides, fatty acids, or waxes [109]. The surface is formed by surfactants or emulsifiers, which stabilize the nanoparticles and prevent aggregation [110]. In recent years, SLNs have been widely employed as drug delivery systems and bioactive food molecules for cancer treatment and other diseases, which offer a better drug solubility, controlled release, enhanced stability, and targeted delivery to specific tissues [91,111,112].

A transdermal emulgel of SLNs based on a pomegranate extract showed potential against Ehrlich ascites carcinoma [111]. A pomegranate extract is rich in phenolic compounds such as flavonoids (catechins, anthocyanins, and other complex flavonoids) and ellagitannins (punicalagin, punicalin, pedunculagin, gallic acid, and ellagic acid), which promote anticancer effects through their antiproliferative and pro-apoptotic characteristics. The application of the transdermal emulgel to the skin of mice reduced tumor development and evolution due to its ability to restrict cell growth. In another study, nanoencapsulated curcumin in SLNs was more effective against A549 lung cancer cell lines than the free compound. The systems showed a sustained drug release and increased cellular uptake [112]. Similarly, quercetin-loaded SLNs effectively inhibited the growth of the invasive breast cancer cell line MDA-MB 231, improving bioavailability and intrinsic apoptotic pathways [91]. Thus, SLNs loaded with polyphenols are very promising systems for the delivery of active substances that inhibit tumor growth and may be an alternative for the treatment of several types of cancer.

- Lipid nanoemulsions

Nanoemulsions are colloidal dispersions composed of two immiscible liquids (usually oil and water) stabilized by surfactants or co-surfactants, typically transparent or translucent, thermodynamically stable, and have droplet sizes of 10–200 nm [113]. The small droplet size and high surface area of nanoemulsions offer unique properties and advantages for various applications. Especially, the encapsulation of polyphenols within nanoemulsions can offer several benefits for cancer treatment [114]. The improved solubility and stability provided by nanoemulsions can enhance the bioavailability and efficacy of polyphenols, potentially leading to improved anticancer effects [115].

Chen et al. 2020 [42] produced EGCG-loaded nanoemulsions derived from green tea to evaluate the anticancer effect against human lung cancer. Nano-EGCG improved its stability and promoted anticancer effects against human lung cancer cell lines (H1299 and A549) through the activation of the AMP-activated protein kinase signaling pathway. Nanoemulsions containing an avocado peel extract have also demonstrated anticancer effects against B16F10 melanoma cells [116]. Avocado skin is a rich source of procyanidins, a member of the proanthocyanidin (or condensed tannins) class of flavonoids. These compounds are involved in critical molecular mechanisms related to inflammatory and malignant diseases, including cancer prevention.

#### 3.1.3. Inorganic Nanomaterials

Inorganic nanomaterials have received considerable attention as safe nanoplatforms for cancer therapy due to their unique properties, such as a high stability, versatility, and load capacity [117]. Among the inorganic nanomaterials most used as nanocarriers for tumor cells, gold nanoparticles (AuNPs), mesoporous silica nanoparticles (MSNs), and quantum dots (QDs) stand out [26,57,70]. Furthermore, these functional nanomaterials have offered unique and versatile properties for delivering therapeutic agents, detecting tumors, and providing targeted treatment [118].

- Gold nanoparticles (AuNPs)

AuNPs have become appealing options for improving drug delivery because of their distinct biological and chemical characteristics, such as a targeted delivery, biocompatibility, and an inert and reduced toxicity [119]. In the last decades, AuNPs have been characterized and developed to provide various drugs and natural compounds for cancer [120,121]. In this context, AuNPs are excellent drug carriers protecting the conjugated drug from enzymatic metabolization [122]. Recently, polyphenolic compounds loaded onto AuNPs have been designed for a sustained and targeted delivery for cancer therapies.

**Figure 4:**
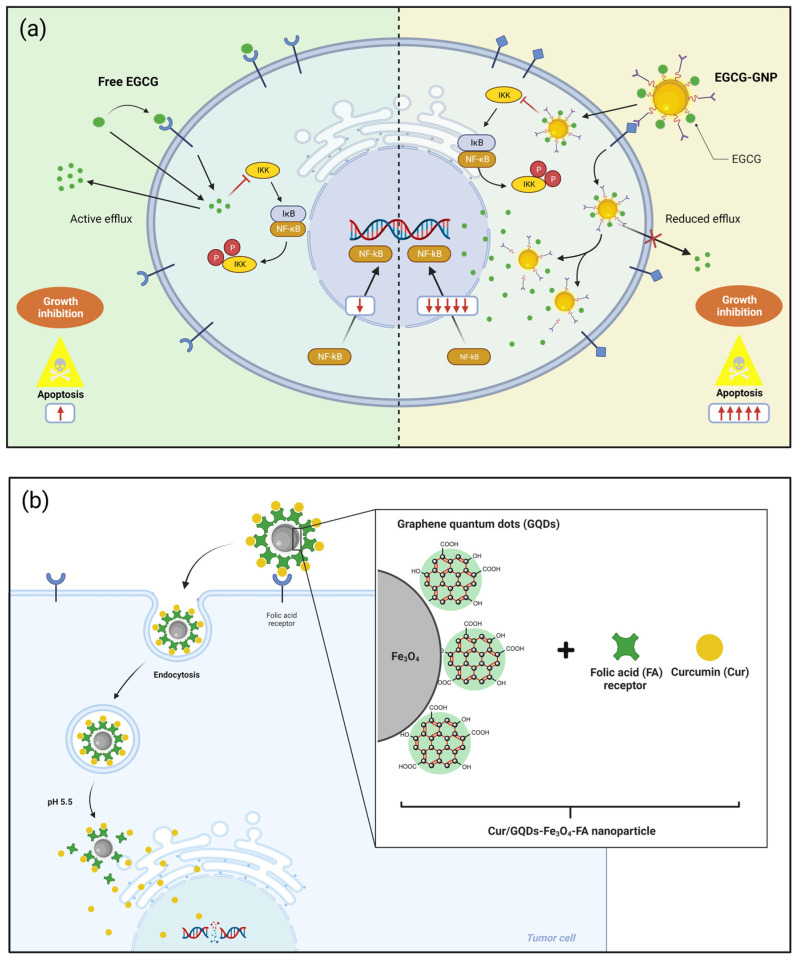
Schematic representation of some polyphenol-loaded delivery systems based on inorganic nanomaterials: (**a**) epigallocatechin-3-gallate (EGCG)-loaded gold nanoparticles (EGCG-GNP) exhibit superior antitumor activity compared to the free polyphenol compound. EGCG-GNP show a sustained drug release over time, inducing more apoptosis in cancer cells than free EGCG. Mechanistically, EGCG-GNP inhibited the nuclear translocation and transcriptional activity of nuclear factor-kappa B (NF-κB) more potently than EGCG, and (**b**) smart pH-responsive magnetic graphene quantum dot (GQD) nanocarriers improve the efficiency of curcumin as a non-toxic and hydrophobic anticancer drug. Folic acid (FA) was conjugated with GQD-Fe_3_O_4_ nanoparticles as a selective nanocarrier targeting agent. An acid medium (pH 5.5) favored a sustained drug delivery, being ideal for cancer-targeted nanocarriers.

In a study by Lee et al. (2022) [26], resveratrol-loaded AuNPs enhanced caspase-mediated apoptosis in pancreatic PANC-1 cells through the intrinsic mitochondrial apoptotic pathway. Resveratrol-AuNPs with a size of 40 nm showed a rapid release rate of resveratrol within 96 h at pH 5.0, demonstrating the possibility of a greater efficiency in delivering the bioactive compound through blood vessels to the tumor. Similarly, resveratrol-conjugated AuNPs also demonstrated a better antitumor efficacy against human breast (MDAMB-231), pancreatic (PANC-1), and prostate (PC-3) cancers [123]. Due to the chemical structure of the polyphenol, resveratrol was used to reduce Au^3+^ to Au^0^, thus producing resveratrol-conjugated AuNPs and encapsulation with gum arabic. Increased resveratrol corona in AuNPs showed superior anticancer effects, while the encapsulation material provided protein matrix support to increase resveratrol loading on the surface of AuNPs. EGCG-loaded AuNPs exhibited superior antitumor activity compared to conventional AuNPs and free compounds [65]. The authors suggested that EGCG-AuNPs could inhibit the nuclear translocation and transcriptional activity of nuclear factor kappa B (NF-κB) in PC3 and MDA-MB-231 cancer cells. These alternatives provide a promising platform for the delivery of polyphenols for cancer therapy (Figure 4a).

- Quantum dots (QDs)

QDs are semiconductor crystals typically spherical- or rod-shaped, with sizes ranging from 2 to 10 nm [124]. In cancer therapy, QDs have been applied explicitly as (i) fluorescent probes to label cancer cells, allowing accurate imaging and detection of tumors; (ii) for a targeted drug delivery, which can specifically target cancer cells; and (iii) in photodynamic therapy, where light activates photosensitizing agents and destroys cancer cells [125,126,127]. Furthermore, natural extracts rich in polyphenolic compounds have recently been used to synthesize QD-based nanomaterials for bioimaging and cancer cell therapy applications [128,129].

Lately, QDs have been functionalized with polyphenols or loaded with polyphenol-containing nanoparticles to treat various tumor cells. Metal–organic frameworks (MOFs) and graphene-based QDs were recently prepared for the delivery of curcumin for cancer therapy [57]. Nanostructures were coated with chitosan to provide a greater biocompatibility. The nanocarrier showed a pH-sensitive swelling behavior and released 38.3% of curcumin in an acid medium (pH 5.0), enabling a better curcumin bioavailability for cancer treatment. Compared to the control group, nanostructures of curcumin-loaded QDs exhibited more significant cytotoxicity against MDA-MB 231 breast cancer cell lines. The results showed that nanomaterials based on curcumin-loaded QDs might be a potential candidate for use as a biocompatible carrier with a controlled drug release capacity.

Similarly, nanocarried curcumin from QDs prepared with graphene oxide (GO) was applied against two cancer cell lines, MCF-7 (human breast cancer cell line) and MG-63 (osteosarcoma cell line) [130]. The QD-based nanocarrier was conjugated with folic acid to ensure targeting in cancer cells. The QD nanocarrier showed superparamagnetic behavior, which shows that an applied magnetic field can direct it to the cancerous tumor site without damaging healthy tissues. Like the previous study, curcumin showed better cumulative release results in an acid medium (33% at pH 5.5). The in vitro cytotoxicity data showed that cancer cells were more vulnerable to the conjugated nanocarrier, which revealed the selective role of folic acid as an active targeting agent (Figure 4b).

- Mesoporous silica nanoparticles (MSNs)

MSNs are functional nanomaterials, generally in the 10 to 100 nm range, and composed of a porous silica structure with regular pore structures [131]. These materials have a high specific surface area, high pore volume, and adjustable pore size, which makes them suitable for various applications such as drug delivery [132]. Lately, MSNs have gained prominence in cancer therapy due to their ability to encapsulate drugs and bioactive compounds in a controlled manner [133]. Polyphenolic compounds such as curcumin, resveratrol, and EGCG have been nanoencapsulated in MSNs for cancer therapy [133,134].

Recently, Sailor et al. (2021) [70] evaluated the potential of resveratrol-loaded MSNs to treat human melanoma. The spherical MSN systems (sizes of 60 nm) showed a high encapsulation efficiency (>93%), which promoted the amorphization of resveratrol, increasing the in vitro release in an acid medium (pH 5.2), an environment more conducive to tumor growth. In vitro cytotoxicity studies against different human melanoma cell lines (A375 and MNT-1) showed a decrease in cell viability with increasing concentrations of resveratrol-loaded MSNs, indicating the potent action of the released resveratrol on both cell lines. Similarly, various arrays of functionalized MSNs were used to encapsulate resveratrol to increase its solubility and bioavailability against lung (A549) and breast (MDA-MB-231) cancer cell lines [134]. All materials containing silica carriers increased the resveratrol dissolution rate. In particular, MSN systems functionalized with propanethiol and isocyanate exhibited the most excellent anticancer effects against A549 cells (IC_50_ = 26.06 and 36.5 µg/mL, respectively) and MDA-MB-231 (IC_50_ = 35.56 and 19.30 µg/mL, respectively), which highlights their potential use against cancer.

Curcumin-laden MSNs have also been extensively investigated in breast cancer therapy. For example, in a study by Mohebian et al. (2023) [135], the investigation of the cytotoxicity of curcumin-loaded amine-functionalized MSN nanostructures showed that even at low concentrations, there was a decrease in the viability of MCF-7 cells compared to free curcumin after 72 h. This effect was further confirmed by studying cellular uptake using confocal fluorescence microscopy. Furthermore, the authors reported that curcumin-loaded MSNs affected mRNA and protein levels of Bax, Bcl-2, caspase-3, caspase-9, and hTERT relative to curcumin treatment. Thus, the authors suggest that MSNs may be a promising alternative for curcumin loading and safe therapy against breast cancer.

#### 3.1.4. Carbon-Based Nanomaterials

In this class of nanocarriers, carbon nanotubes (CNTs), graphene and its derivatives, including graphene oxide (GO) and reduced graphene oxide (rGO), and fullerene have gained a greater prominence for cancer nanotherapy in recent years [136]. These materials have been widely explored as drug nanocarriers and natural compounds applied in treating cancer cells, in photothermal medicine, and as cancer detection and imaging agents [137,138].

- Carbon nanotubes (CNTs)

In recent years, CNTs and their derivatives have gained a great prominence in cancer therapy, mainly applied as platforms for a targeted drug delivery, photothermal therapy, imaging and diagnoses, and combination therapy [139,140]. Their unique properties, including a high surface area, tunable surface chemistry, and excellent mechanical strength, make them attractive for cancer therapy applications [141].

Multi-walled carbon nanotubes (MWCNTs) and carboxymethylcellulose (CMC) have recently been prepared as a novel curcumin oral delivery system for treating colon-specific cancer [27]. Initially, curcumin loaded in MWCNTs/CMC showed a drug release rate of 11.6% and 76.5% at pH 1.2 and 7.4, respectively. This indicates that MWCNTs/CMC/curcumin has the potential as a cancer-drug-controlled delivery system, which can inhibit tumor growth in an acidic environment. Cell viability assays confirmed the antitumor activity of the delivery system against SW-480 human colorectal adenocarcinoma cells, showing cytotoxic potential at a high concentration (IC_50_ = 752 μg/mL).

**Figure 5:**
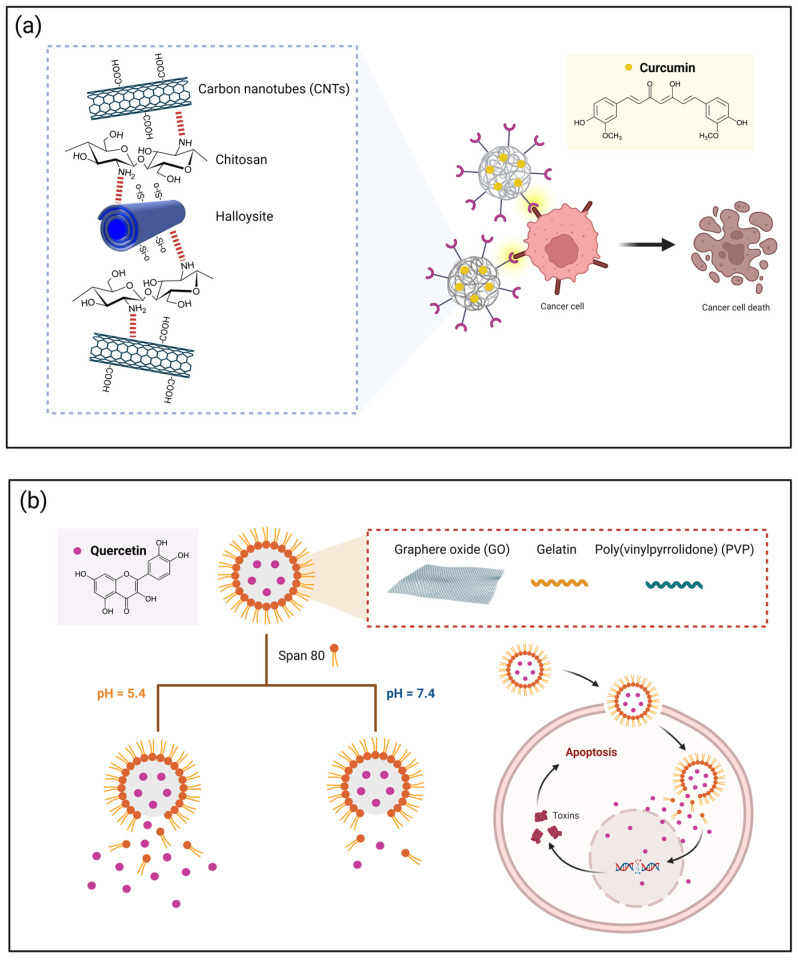
Schematic representation of some polyphenol-loaded delivery systems based on carbon nanomaterials: (**a**) chitosan/halloysite/carbon nanotube (CNT) nanocarrier for the pH-sensitive delivery of curcumin to cancerous media. The controlled and sustained release of the drug in an acid medium promotes the induction of apoptosis in cancer cells, with an improved cytotoxicity for the curcumin-loaded nanocarrier compared to free curcumin, and (**b**) graphene oxide (GO) nanocarriers coated with pH-sensitive gelatin-poly(vinylpyrrolidone) (PVP) to enhance quercetin delivery. After forming the GO-gelatin-PVP nanocomposite and drug loading, a nanoemulsion was obtained as a sustained and targeted release system. The acidic pH of 5.5 promoted a greater drug release, which may favor the imbalance of the tumor microenvironment, causing cell death by apoptosis.

Similarly, a chitosan/halloysite/carbon nanotube (CNT) nanocarrier has been employed to deliver curcumin as a pH-sensitive delivery system for cancer [142]. The drug release results showed that curcumin has a sustained and controlled release profile up to 48 h, mainly at pH 5.4, which simulates the tumor environment. After 96 h, 92% and 96% release rates were found at pH 5.4 and 7.4, respectively. Regarding in vitro cytotoxic tests, the curcumin-loaded nanostructure showed the induction of apoptosis in MCF-7 (breast cancer) cells and exhibited an enhanced cytotoxicity of the drug-loaded nanocomposite compared to free curcumin. The authors suggested that curcumin-loaded chitosan/halloysite/CNT nanocarriers may be a good option for drug delivery systems, particularly breast cancer treatment (Figure 5a).

- Graphene

Graphene and graphene-based nanomaterials have attracted significant attention in anticancer drug delivery, mainly based on natural bioactive molecules [20,143]. Graphene is a two-dimensional carbon allotrope with unique properties, such as a large surface area, excellent mechanical strength, and high electrical and thermal conductivity [144,145]. These characteristics make graphene an ideal platform for developing nanomaterials for drug delivery. Among the main graphene-based delivery systems for delivering polyphenols in cancer, the following stand out: graphene oxide (GO) [146], reduced graphene oxide (rGO) [147], graphene quantum dots (GQDs) [148], graphene nanoribbons [149], and graphene-based nanocarriers [150].

Recent research shows that graphene-based nanomaterials are a trend toward developing efficient materials for cancer therapy. Najafabadi et al. (2023) [20] recently investigated gelatin/PVP-coated GO nanocarriers for the targeted delivery of quercetin against MCF-7 breast cancer cells. The resulting pH-sensitive drug delivery system showed an encapsulation efficiency of 87.5% and a zeta potential of −40 mV, indicating a good stability of the nanocarriers. The kinetic release profile of quercetin from the nanocarriers showed a controlled and sustained release of the drug in the long term, with a drug release rate after 96 h of 95.5% and 91% at pH 5.4 and 7.4, respectively. This was improved due to the dual nanoemulsion, resulting in a better drug entrapment efficiency. Cytotoxicity data revealed that the nanocarrier promoted the death of 53.14% of cancer cells, with a rate of 36.51% in the apoptotic phase (Figure 5b).

Another study investigated the anticancer potential of a quercetin-loaded GO/iron oxide/AuNP nanocomposite in MCF-7 breast cancer cell models [151]. A significant increase in the cytotoxicity of the quercetin-loaded nanocomposite (IC50 = 42.32 µg mL^−1^) against the breast cancer cell line was observed, compared to the free quercetin. Meanwhile, the quercetin-free nanocomposite did not show a significant toxicity against cell lines, proving that the observed toxicity of the quercetin-loaded nanocomposite was caused by the drug released from the nanocomposite [151].

The effect of a curcumin-loaded GO nanocomposite on cell membrane integrity and possible redox changes in colon cancer cells (HT-29 and SW-948) was investigated by Al-Ani et al. (2020) [146]. Using transmission electron microscopy (TEM) images, the authors showed that sheets of nanocomposites were captured inside cancer cells with endocytosis, forming endosomes. Microscopic observations also confirmed the presence of apoptotic bodies with chromatin condensation in both colon cancer cells. Furthermore, redox parameters revealed a time-dependent increase in ROS accompanied by regressive levels of GSH and SOD (superoxide dismutase). Thus, the curcumin-loaded GO nanoformulation was considered to have the potential for understanding novel mechanisms for cancer therapy.

### 3.2. Polyphenol Co-Delivery Systems

Cancer cell treatment can be performed using co-delivery systems containing a hybrid mixture of polyphenols [152]. Polyphenol co-delivery systems are commonly applied carriers for releasing active substances from different sources, enhancing the anticancer activity and antiproliferative effect [153].

Different combinations of polyphenols have been applied in co-delivery systems. Recently, the synergistic delivery of curcumin–resveratrol from dendrimer polymer nanoparticles showed slow-release kinetics over time, which improved their therapeutic effect against neuroblastoma cell lines (SH-SY5Y) [154]. The authors reported that the synergism between curcumin incorporated into the dendrimer and resveratrol induced an increased mitochondrial disruption, affecting the intracellular calcium release and causing cancer cell death. This effect was more pronounced on charged NPs than freely dissolved polyphenols or amphiphilic dendrimer NPs incorporating curcumin or resveratrol separately. In another study, the combination of curcumin–resveratrol loaded from SLN demonstrated a high potential for the synergistic inhibition of highly aggressive melanoma cells [155]. The topical delivery system loaded with the polyphenols showed a better inhibition effect against SK-MEL-28 human melanoma cells (IC_50_ = 1.6 μg/mL) than the free compounds (IC_50_ for curcumin: 6.38 μg/mL; IC_50_ for resveratrol: 3.67 μg/mL).

The synergism of curcumin and chrysin in electrospun nanofibers also showed antiproliferative effects for local breast cancer prevention [156]. Nanofibers loaded with curcumin and chrysin exhibited a better synergistic cytotoxicity against T47D cancer cells than the free compounds. A gene expression analysis showed that drug-loaded nanofibers decreased mRNA expression levels of apoptotic (Bax, Bcl-2, caspase-3, and caspase-7) and anti-apoptotic genes, including Cyclin D1, hTERT, and Bcl-2. Thus, nanofibers loaded with curcumin and chrysin may provide a promising vision to prevent the local recurrence of breast cancer [156].

In most cases, the combination of two polyphenols usually contributes synergistically to enhance the anticancer effect. However, in a study by Piwowarczyk et al. (2022) [157], an antagonistic effect of curcumin with a peracetylated derivative of (−)-epigallocatechin 3-O-gallate (pEGCG) in a liposomal nanoformulation was observed in the treatment of tumor cells. Research results showed that combining curcumin with pEGCG in the tested concentration range decreased curcumin activity in 5637 (human bladder grade II carcinoma) and LNCaP (human prostate carcinoma) cells. Meanwhile, the sequential administration of these nanoencapsulated compounds increased cell death via apoptosis compared to treatment with the free compound [157].

Although the study of novel polyphenol co-delivery systems has made significant progress in in vitro tumor cell treatment, the scientific literature still lacks more in vivo research. Therefore, it is essential to dedicate more research efforts toward comprehending the toxicological impacts of combining polyphenols in co-delivery systems to advance both research and clinical treatment.

### 3.3. Polyphenol–Drug Delivery Systems

As seen so far, polyphenol delivery systems have been widely investigated for cancer therapy in recent years. However, nanotechnology research has gone deeper and deeper to overcome conventional cancer therapy’s main challenges, such as a poor drug solubility, limited drug stability, systemic toxicity, and inefficient tumor targeting. With that in mind, combining polyphenol-based bioactive compounds and anticancer drugs may offer a potential synergistic approach to cancer therapy [158]. Thus, polyphenol–drug delivery systems have lately been designed to increase the effectiveness of anticancer drugs by improving their delivery to tumor cells and minimizing their distribution to healthy tissues, thus reducing the drug side effects [159].

Recently, niosomal nanoformulations loaded with tamoxifen and curcumin revealed a high anticancer potential against MCF-7 breast cancer cells [160]. Drug-loaded niosomes caused the upregulation of the Bax and p53 genes and downregulation of the Bcl2 gene. Flow cytometry studies revealed that drug-loaded niosomes increased the apoptosis rate in MCF-7 cells compared to the combination of tamoxifen and curcumin due to the synergistic effect between the two drugs and increased cellular uptake by the niosomal formulation. The results indicated that the release of both drugs from niosomal carriers is controlled, which may reduce the side effects of the drugs in normal cells [160].

The addition of gallic acid (GA), a polyphenolic compound that can be found in various natural foods and fruits, together with doxorubicin (DOX) in multi-crosslinking nanocapsules increased the cytotoxic effect for HepG2 liver cancer cells [161]. Results indicated that the systems could minimize side effects and optimize cancer therapeutic efficacy. Similarly, adding green tea polyphenolic compounds and DOX enhanced the anticancer effect of a enzyme-responsive bovine serum (SBA) nanocarrier against different cancer cell lines (MCF-7, HELF, and MCF10) [158]. The results showed that the application of the nanocarrier could reduce the toxic side effects of DOX, increase the cellular targeting uptake, and perform nuclear drug delivery [158].

The antiproliferative, cell cycle arresting, and apoptotic effect of niosomes loaded with curcumin and artemisinin against SW480 human colon cancer cells was recently investigated by Amandi et al. (2023) [162]. Although artemisinin is widely used in the treatment of malaria, several studies indicate that the drug has shown cytotoxic and anticancer properties against several types of cancer. In the study, the authors confirmed that the curcumin–artemisinin combination in the niosomal formulation showed a significantly higher toxicity (IC_50_ = 3 µM) against SW480 cells compared to the respective drug formulations (IC_50_ = 10 µM and 9 µM for curcumin and artemisinin niosomes, respectively) or their free compounds (IC_50_ = 16 µM and 10 µM for curcumin and artemisinin, respectively). Furthermore, the formulation increased the expression of Bax, Fas, and p53 genes and reduced the expression of Cyclin D1, hTERT, Survivin, Rbp, and Bcl2 genes as pro-apoptotic and cell-cycle-associated factors [162].

In a study by Mogheri et al. (2021) [163], the anticancer effect of metformin- and silibinin-loaded PLGA-PEG nanoparticles synergistically improved chemotherapy in human non-small cell lung cancer A549 cells. Although metformin was originally an antidiabetic drug, it has also been used to prevent cancer incidence. Together with silibinin, a natural polyphenolic flavonoid, the nanoencapsulated drugs prevented A549 cancer cell proliferation and induced apoptosis by upregulating caspase-3, caspase-7, and Bax, and downregulating Bcl-2, hTERT, and Cyclin D1. Thus, therapy with drug–polyphenol-loaded nanoparticles may be an attractive strategy based on herbal substances for treating lung cancer [163].

## 4. Cancer Models in Preclinical Studies

The effect of polyphenol delivery systems on various cancer models has been investigated in preclinical studies using animals. In general, the studies evaluated the therapeutic efficacy of the polyphenol delivery system or systems combined with chemotherapeutics against animal models xenografted with tumor cells [66,164]. Furthermore, some studies performed a toxicological analysis against non-target organs to ensure the safety of the formulations [165,166,167]. Among the main types of cancer, we highlight breast, prostate, lung, colorectal, and cervical cancers as the most investigated models in recent years.

Table 3 shows some complementary studies that evaluated the efficiency of polyphenol-loaded nanosystems for treating different types of cancer in animal models.

### 4.1. Breast Cancer

Polymeric nanoparticles were explored as potential nanocarriers of polyphenolic molecules applied in in vivo studies to treat breast cancer. Feng et al. (2019) [177] developed fisetin-loaded PLA nanoparticles (PLA-Fis NPs) to increase therapeutic efficacy for treating breast carcinoma induced by 4T1 cells. Animal experiments showed that spherical-shaped nanoparticles had a longer half-life (3.42 h) compared to the free flavonoid (1.82 h), which was related to greater control over the release of the compound from the delivery system. According to the authors, the negative charge of PLA-Fis NPs (−15.63 mV) offered a possibility of escaping the undesirable recognition from other opsonins or the capture from the mononuclear phagocyte system. Similarly, Jiang et al. (2020) [166] observed suitable toxicological safety when applying PEG-PCL nanoparticles loaded with flavonoid silibinin (SLB-NPs) to treat BALB/c mice induced with breast cancer. In that study, few changes in animal weight and non-target organs were observed after treatment with the nanoformulation. The nanoparticles with a mean diameter of 37.2 nm promoted a decrease of up to four times in the tumor volume after 22 days of treatment. Furthermore, the study revealed a reduction in tumor-associated fibroblast cells (TAFs) compared to the control group and 18% more detection of apoptotic cells than this group.

**Figure 6:**
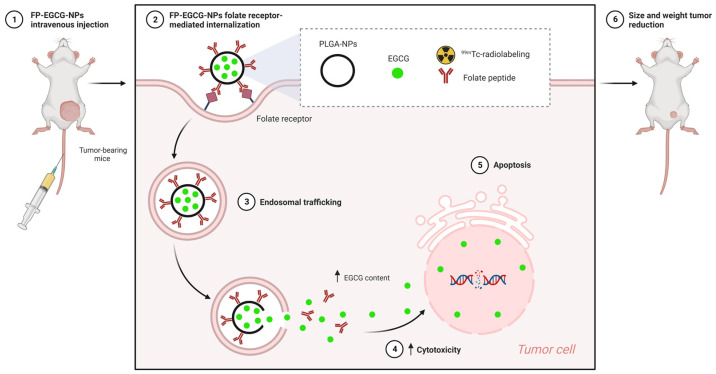
Schematic representation of the anticancer effect of a folate-decorated epigallocatechin-3-gallate (EGCG)-loaded nanoformulation in breast cancer xenograft mice models: the EGCG-loaded formulation is injected intravenously into mice with a xenografted breast tumor (1); the folate receptor on the surface of nanoparticles favors endocytosis due to its ability to bind to other receptors on tumor cells (2, 3); the targeted release of EGCG enhances the cytotoxic effect on the tumor cell (4), resulting in apoptosis and cell death (5), which reduces tumor size and weight (6).

PLGA nanoparticles anchored with folate peptides loaded with EGCG proved to be potential candidates for actively targeting breast cancer drugs [178]. The study showed that polymer nanoparticles with a mean diameter of 169 nm (PDI = 0.063) controlled the release of EGCG for up to 48 h in the plasma of animals with breast tumors induced by MDA-MB-231 cells. This effect was favored by the structure of the nanoparticles composed of the folate receptor, improving endocytosis and the internalization of polyphenol in tumor cells, which promoted tumor inhibition without affecting healthy organs. This was further proven by a technetium-99m (^99m^Tc) radiolabeling study, which showed the increased accumulation of EGCG-loaded nanoparticles in liver tissue and urinary excretion (Figure 6).

Some studies have proposed using polymer micelles as nanocarriers of natural polyphenolic agents for treating breast cancer. Among them, Lee et al. (2021) [179] developed mPEG-PLA nanoparticles conjugated with aspirin to carry the chalcone derivative DK143. Nanoparticles with a mean diameter of 22.55 nm (PDI = 0.113) promoted a significant reduction in tumor growth after 30 days of treatment, without toxicity in organs such as the heart, lungs, spleen, kidneys, and liver, revealing the selectivity of nanoparticles against cell tumors. Eskandari et al. (2021) [62] prepared a nanoformulation of PLGA and a levan polysaccharide for the combined treatment of curcumin and the chemotherapeutic gemcitabine against breast cancer induced by MAT B III cells. This study showed that treatment with the nanoformulation reduced the tumor size approximately 405 times compared to the control group treated only with chemotherapy, demonstrating the success of using polyphenolic agents in treating breast carcinoma in vivo.

Other types of nanoparticles are also described as nanocarriers of phenolic compounds used as therapeutic agents against breast cancer induced by 4T1 cells. For example, Yilmaz et al. (2019) [180] prepared a hybrid platform of AuNPs coupled to the macrocycle compound calixarene, which contained quercetin as a polyphenolic therapeutic agent. After 22 days of treatment, the animals showed a 30% inhibition of tumor growth compared to the control group and an increase in the expression of apoptosis and pro-apoptotic regulatory genes, suggesting cell death via apoptosis mediated by the release of polyphenol. In another study, Zhao et al. (2020) [167] showed that applying albumin nanoparticles loaded with resveratrol (average diameter ranging from 160 to 180 nm) can be a safe alternative for treating breast cancer. The authors showed that nanoparticle treatment reduced the tumor volume without systemic toxicity or the impairment of liver and kidney functions. Furthermore, histological analyses revealed necrosis in the tissues of animals treated with resveratrol-loaded nanoparticles without inflammation or lesions in non-target organs, indicating low adverse effects.

### 4.2. Prostate Cancer

Resveratrol-loaded SLNs were recently applied to treat mice’s prostate cancer induced by PC3 cells [181]. The systems presented the encapsulation efficiency of 74.67% of the polyphenolic compound, in addition to an average diameter of 126.85 nm and zeta potential of around −24.23 mV. The SLNs showed the controlled release of the polyphenolic compound after 48 h in the animal’s body, with a half-life of 8.22 h and a 7.56 times greater accumulation in the prostate. The results were superior to the free compound, indicating that resveratrol-loaded SLNs are potential systems for tumor-site-specific targeting.

Some studies used aptamer oligonucleotides to functionalize the surface of nanoparticles to improve the specificity for the treatment of prostate cancer. For example, Chen et al. (2020) [182] explored a hybrid nanoparticle based on a PEG-PLGA core and a lipid shell functionalized with aptamer A10-3.2 for loading curcumin. The authors observed, from in vivo experiments, that the nanoformulation loaded with 82.6% curcumin promoted a significant decrease in the tumor volume of mice with prostatic tumors induced by LNCaP. Furthermore, no changes in the body weight or kidney and liver organs were observed after treatment. In another study, Ma et al. (2019) [183] functionalized the surface of curcumin-loaded nanoliposomes with aptamer A15. Nanoliposomes containing 92.3% curcumin showed the considerable inhibition of prostate tumors induced by DU145 cells compared to the control group, suggesting a specific delivery of curcumin to the tumor site.

Recently, PEGylated surface-coated PLGA nanocapsules were developed to deliver quercetin to treat prostate cancer [184]. Nanocapsules with a particle diameter of 130 nm showed more significant accumulation at the tumor site induced by PC3 cells, promoting a decrease in the tumor volume after treatment, with a tumor inhibition rate of 50.57%. The authors also reported that some areas of necrosis in the tissues treated with the nanoparticles were observed, but no pathological lesions were visualized in tissues of non-target organs. Similar results were reported by Zhang et al. (2022) [185] when applying PEGylated nanoliposomes to deliver resveratrol to tumors induced by PC3 cells. Treatment with nanoliposomes (particle diameter of 103.39 nm) showed that even after 42 days, there was no change in the body weight of the animals. On the other hand, the nanosystems induced chromatin dissolution, with cell death by apoptosis, in addition to a decrease in the expression of Ki-67 cell proliferation proteins.

Nagesh et al. (2019) [186] evaluated the antitumor effect of the combination of tannin and nanoencapsulated docetaxel for tumor treatment in animals with PC3-cell-induced prostate cancer. Nanoparticles with a size of 65.47 nm showed more significant accumulation at the tumor site and regression of tumor growth after 4 weeks of treatment. The results showed that tumor growth and weight were lower compared to the control group (no treatment) after 72 h. Furthermore, apoptotic signaling with antisenescence capability was observed in the excised tumor tissues without significant liver and kidney toxicity. Similarly, the synergistic effect of EGCG with docetaxel was also investigated in a study by Chen et al. (2020) [187]. The authors developed a multifunctional polysaccharide-based nanoparticle conjugated with vitamin E and hyaluronic acid to treat PC3 cell tumors in mice. A significant reduction in tumor volume, probable cell death by necrosis, and no toxicity to the other organs of the animals were observed, evidencing an adequate therapeutic specificity. Moreover, immune–histochemical assays showed a decrease in the expression of the Ki-67 protein of cell proliferation and an increase in the expression of M30 related to apoptosis.

### 4.3. Lung Cancer

Nanomaterial-based polymeric carriers have been extensively employed to treat lung cancer induced by A549 cells. For example, studies by Baksi et al. (2018) [188] and Wang et al. (2021) [189] demonstrated that quercetin-loaded chitosan nanoparticles showed similar results regarding the mean particle size (100 nm and 300 nm), zeta potential (18.03 mV and 23.6 mV), and encapsulation efficiency (79.78% and 87.73%). In vivo assays showed that nanoparticles were good candidates for specific targeting, significantly reducing the volume and weight of tumors extracted from animals. Furthermore, the nanoparticles did not cause any apparent damage to normal organ tissues, indicating a low systemic toxicity and good tolerance to treatments with the quercetin-loaded nanoparticles. In turn, Huo et al. (2020) [190] produced dextran-based nanoparticles for the controlled delivery of silybin and paclitaxel against a tumor model of lung cancer in mice. Nanoencapsulated systems containing 69.6% of a polyphenolic compound showed a significant increase in the half-life time (5.0 h) compared to its free form (1.31 h). Regarding the anticancer effect, the systems promoted a substantial inhibition of tumor growth, an increase in cells undergoing apoptosis, and a decrease in TAF cells.

In a study by Song et al. (2018) [191], lipid and polymeric nanoparticles (LPNs) loaded with resveratrol showed high anticancer potential against lung cancer induced by HCC827 cells. The nanoformulation reduced the tumor volume twice compared to the control group (treated with free polyphenol), showing a tumor inhibition rate between 35% and 40% without changing the weight of the animals. In another study, similar nanostructures loaded with curcumin and coated with polysaccharides were produced by Xie et al. (2020) [104]. After evaluating the encapsulation of the polyphenolic molecule (79.23%), the authors reported that the plasmatic concentration and biodistribution of the nanoparticles in the animals’ organisms were more significant than their free form, with the volume and tumor mass as 58.39% and 59.7%, respectively, which were lower when compared to the control.

Riaz et al. (2019) [192] prepared functionalized nanoliposomes with the T7 peptide for quercetin loading and evaluated their in vivo efficacy against lung tumors induced by A549-luc cells. The authors described an efficiency of 95% in encapsulating the flavonoid and, through biodistribution assays, reported a specific accumulation of nanoliposomes in the affected organ during 96 h of treatment. Moreover, the inhibition of tumor growth and survival of up to 69 days was observed in animals treated with the nanoformulation after pulmonary injection. In a study by Song et al. (2020) [193], MSNs modified with folic acid were applied as myricetin flavonoid delivery systems. After 27 days of treatment, tumors induced by A549 cells showed a considerable increase in the percentage of apoptotic cells and a significant accumulation of nanoparticles at the tumor site, in addition to significant reductions in the tumor size, volume, and weight. Furthermore, the flavonoid-loaded systems promoted a decrease in Ki-67 protein expression related to cell proliferation, no histopathological alteration, and serum levels of liver and kidney markers. Thus, various polyphenol delivery systems can be effectively applied to treat lung cancer.

### 4.4. Colorectal Cancer

Colorectal cancer is one of the most prevalent and deadly malignancies worldwide [194]. Despite advancements in therapeutic strategies, developing effective treatments remains a significant challenge [195]. Thus, several innovative polyphenol delivery systems have been investigated in in vivo studies to improve the therapeutic outcomes of colorectal cancer treatment.

Chaurasia et al. (2018) [196] synthesized a nanosystem based on the polymer Eudragit^®^ E100 loaded with the flavonoid naringenin to treat colorectal cancer induced by CRC cells. The nanosystem presented an encapsulation capacity of 68.83% and a particle size of 430.42 nm. The authors observed that the plasmatic concentration of the nanoencapsulated polyphenol was about 88 times higher compared to the free compound, with a half-life of 4.298 h (nanoencapsulated naringenin) and 2.6323 h (free naringenin), in addition to a smaller tumor volume and lower mean weight of animals treated with the nanoformulation.

In a study by Zhang et al. (2022) [197], PCL-PEG nanoparticles loaded with resveratrol showed an encapsulation efficiency of 45.25% and an average particle size of 160.91 nm. With a negative surface charge (−31.64 mV), the retention of nanoparticles in the animals’ blood for 48 h was reported. Additionally, the authors mentioned that no pathological alteration in the tissues of mice with colorectal cancer induced by HT29 cells was observed. However, there was an increase in ROS production and a decrease in Ki-67 protein expression after treatment with the nanoparticles. Regarding colon tumors induced by HT29 in mice, a study by Sem et al. (2019) [198] revealed lower tumor growth and a decrease in blood vessels (inhibition of angiogenesis), levels of expression of the CD-31 protein (responsible for vascularization), and Ki-67 (cell proliferation marker) after treatment with liposomes loaded with the flavonoid apigenin.

Curcumin, a polyphenol with hydrophobic characteristics and a low bioavailability, has been used in some studies as a bioactive molecule for the in vivo treatment of colorectal cancer. For example, Karabasz et al. (2019) [80] developed highly stable alginate–curcumin polymer micelles (160 nm particle size) (−53 mV zeta potential) for the treatment of colorectal tumors. The average weight of tumors after 30 days of treatment with nanocarriers was lower than the control group. The analysis of the serum cytokine profile showed lower concentrations of pro-inflammatory ones (IL-23, IL-1α), reflecting a positive aspect of the presence of curcumin due to its ability to maintain the activity of the immune system at a low baseline level. Similarly, curcumin was also addressed in a study by Liu et al. (2022) [199], where a nanoplatform coated with manganese dioxide (MnO_2_) and BSA was built to load doxorubicin and curcumin, resulting in a smaller volume and weight of tumors after treatment. The immune responses of the animals in the group treated with the nanoparticles revealed an increase in the levels of CD^8+^ and CD^4+^ lymphocytes, indicating an immunostimulatory effect; in addition, these nanoparticles positively regulated the serum level of pro-inflammatory cytokines and stimulated antitumor peripheral immunity after reintroduction studies of the tumor.

Quercetin has also been a polyphenolic compound widely applied in colorectal cancer therapy. In a study by Mishra et al. (2020) [200], MSN functionalized with folic acid and magnetite nanoparticles (Fe_3_O_4_) was manufactured as quercetin transporters for tumor targeting and treatment of CT26-induced colorectal cancer in BALB/c mice. The results showed no death of the animals after the treatment period with nanoparticles loaded with quercetin. Otherwise, only 20% of animals treated with free quercetin remained alive after 30 days. These results are consistent since the proliferation of apoptotic tumor cells, such as Ki-67, was low, confirming the ability of such nanostructures to serve as contrast agents, accumulating iron in tumor tissues and promoting a targeted delivery of the biological therapeutic agent. In another study, Liu et al. (2022) [164] developed quercetin-loaded NLCs to treat colorectal cancer from a CRC cell xenograft. The nanosystems had a particle size of 116.8 nm, surface charge of −23.5 mV, and encapsulation efficiency of 83.2%. Briefly, the authors observed that the tumor volume after the injection of quercetin-NLCs was two times smaller than the control group (saline solution) and that, in addition to the weight of the animals not being changed considerably in the group with the nanoformulation, the serum level values of renal and hepatic function (creatinine and ALT), as well as the number of white blood cells (WBC), were also unchanged, indicating in vivo biosafety without systemic toxicity.

### 4.5. Cervical Cancer

Several types of nanosystems have been recently investigated for their ability to carry polyphenolic molecules with therapeutic activity to treat cervical cancer. Zheng et al. (2015) [201] synthesized metal–organic frameworks (MOFs) of imidazole zeolite as potential nanocarriers for curcumin. The authors obtained dodecahedral-shaped nanoparticles with sizes of 119.3 nm and an encapsulation efficiency of 88.2%. Treatment of mice with curcumin-loaded nanoparticles reduced the volume of tumors induced by U14 cells, with a tumor inhibition rate of 85% and cell death predominantly by necrosis. Similarly, Chen et al. (2020) [202] developed EGCG-loaded MOF nanostructures to treat cervical cancer induced by HeLa cells. The authors reported about a 50% efficiency of polyphenol encapsulation in nanoparticles that, in turn, had rounded edges with a diameter of 200 nm and a positive surface charge of 21.9 mV. In vivo autophagy processes were observed with more apoptotic cells in tumor tissues after treatment with EGCG-loaded MOFs. Moreover, the authors reported a decrease of approximately 52.66% in the size of tumors and a low systemic toxicity in renal and hepatic function enzymes.

Zhang et al. (2015) [203] produced PCL nanoparticles as a potential nanocarrier of the isoflavonoid genistein for treating cervical cancer. Spherical nanoparticles with a size of 170 nm, with a negative surface charge (−14.7 mV), showed an encapsulation efficiency of 95.56%. Using the HeLa cell line, the authors induced the growth of cervical tumors in female mice and observed less tumor growth in animals with nanoparticles loaded with genistein. The low toxicity of the polyphenol release system was confirmed by the unchanged body weight of the animals, increasing its anticancer activity. In a study by Zaman et al. (2016) [204], curcumin-loaded PLGA nanoparticles demonstrated advances in the treatment of cervical cancer induced in animals by Caski cells. The authors observed a decrease in the tumor volume, in addition to the suppression of oncogenic proteins, cell proliferation markers, and the regulation of miRNA expression after treatment with nanoparticles.

**Figure 7:**
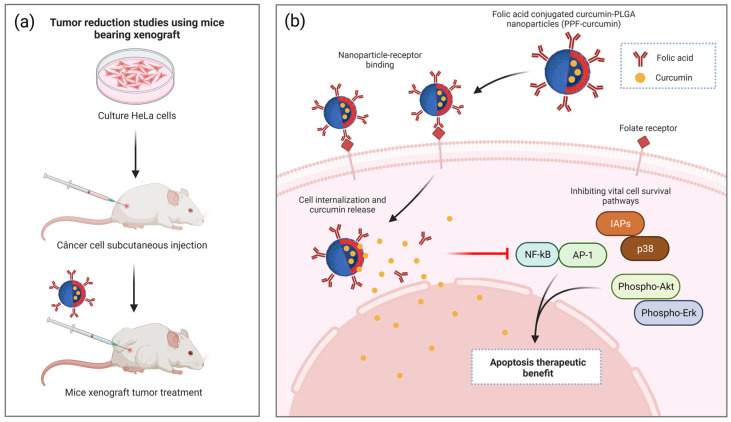
(**a**) Scheme of the treatment of a cervical tumor induced by HeLa cells in xenograft mice using folic-acid-conjugated curcumin-PLGA nanoparticles (PPF-curcumin), and (**b**) possible mechanism of action of PPF-curcumin nanoparticles in the chemosensitization of cervical cancer cells.

Cervical tumors caused by HeLa cells have also been treated with curcumin-encapsulated PLGA-PEG nanoparticles and conjugated with folic acid (Figure 7a) [205]. The acute and chronic toxicity of the nanostructures, evaluated with histology of the animals’ livers, did not reveal signs of liver damage. Curcumin-loaded polymer nanoparticles significantly decreased the tumor volume compared to the control group. This effect was observed due to better targeting and the more excellent retention of polyphenols in the intratumoral region, causing prolonged circulation with greater internalization in cells, which confirms their effectiveness as a chemosensitizing agent. The proposed mechanism of action by which folic-acid-conjugated curcumin-loaded nanoparticles cause the suppression of survival signals, which leads to the chemosensitization of cervical cancer cells, is illustrated in Figure 7b.

The effect of the nanocarrier quercetin on different delivery systems was also discussed in the studies by Luo et al. (2016) [206] and Li et al. (2017) [207]. Both authors investigated the in vivo potential of a quercetin-loaded polymer and liposomal nanostructures for cervical cancer therapy. The studies reported that both developed systems promoted a more significant decrease in the tumor weight of the animals when compared to the control groups. The suppression of disease progression was also observed after the stimulation of specific caspases and inhibition of genes involved in cell proliferation, triggering cell death by apoptosis when quercetin-loaded polymer nanostructures were used. Greater targeting of nanoparticles in the tumor region was observed, which protected other organs against toxicity. Furthermore, animals treated with quercetin-loaded liposomes showed necrosis and apoptosis tissue areas.

Although polyphenol-loaded delivery systems have successfully treated several animals with induced tumors, few human clinical trials have been conducted in recent years [208,209,210]. Furthermore, most current clinical studies used the pure polyphenolic compound in an unloaded form [32,211,212]. In our previous study [7], a critical literature review identified that in the last 15 years, few clinical studies have been conducted applying nanoformulations loaded with polyphenols and other bioactive compounds in human cancer treatments. Thus, it is suggested that a further analysis is needed to ensure the safety and efficacy of polyphenol nanocarriers in clinical cancer therapy.

## 5. Conclusions and Future Perspectives

Food-derived polyphenols have been widely recognized as bioactive compounds with chemopreventive potential. Due to their high antioxidant and anti-inflammatory potential, compounds such as quercetin, curcumin, epigallocatechin-3-gallate (EGCG), and resveratrol have shown promising potential as novel herbal medicines for cancer prevention and treatment. The evidence provided by the scientific literature indicates that these compounds can inhibit tumor growth by inducing cell apoptosis in various types of cancer. However, their therapeutic efficacy is limited due to their low bioavailability, resulting from a high metabolism rate, impaired absorption, inactivity of metabolic products, and rapid bodily elimination. Thus, several nanotechnology approaches have been employed to improve the therapeutic effectiveness of polyphenols for cancer.

Our analysis identified and explored several functional nanomaterials currently employed for encapsulating polyphenols for cancer. Notably, the nanoencapsulation of polyphenols in polymeric nanoparticles and lipid-based nanomaterials emerged as a promising strategy to improve the therapeutic efficacy of these bioactive molecules. Polymeric nanoparticles exhibited a superior self-assembly capacity under specific pH conditions, enhanced biodegradability, and sustained drug delivery. Lipid-based nanomaterials, such as liposomes and niosomes, showed potential as nanocarriers due to their non-toxicity and efficient cell penetration, enabling an improved targeted delivery. While inorganic nanoparticles and carbon-based nanomaterials have been explored as polyphenol delivery systems, their use is limited by toxicity concerns and a low polyphenol loading efficiency. These studies underscore the importance of selecting appropriate delivery systems, considering factors such as stability, loading capacity, controlled release, and specific targeting.

In general, in vitro and in vivo studies showed better results in anticancer activity than free compounds, demonstrating the ability of polyphenol delivery systems to improve treatment efficacy and reduce unwanted side effects. Additionally, polyphenol co-delivery systems and polyphenol–drug delivery systems were found to be promising strategies to potentiate the anticancer effect of polyphenols against tumor cells, with fewer side effects of anticancer drugs and a potential coadjutant in chemotherapy and radiotherapy. These strategies effectively protect polyphenols against degradation, increase their bioavailability, and enable specific targeting of tumor cells.

Future opportunities were identified for novel clinical applications of polyphenol delivery systems for cancer treatment. The possibility of selectively targeting cancer cells, increasing therapeutic efficacy, and reducing side effects opens unknown doors to more efficient and personalized therapies. However, it is essential to emphasize the continuous need for research and development to improve these systems further, ensuring their safety and clinical efficacy. Finally, additional studies are needed to expand the knowledge about polyphenol-loaded delivery systems’ pharmacokinetics, mechanisms of action, bioavailability, toxicity, and biocompatibility and validate their in vivo performance.

## Figures and Tables

**Table 1:** Some types of polyphenols and their therapeutic potential against cancer cells.

Polyphenol	Main Class/ Sub-Class	Food Source	Type of Cancer	Therapeutic Effects on Cancer Cells	References
Quercetin	Flavonoids/Flavonols	Apples, raspberry, blackcurrant, blueberry, orange, cherry, grapes, raspberry, cranberry, strawberry, and green vegetables	Breast	- A significant difference in tumor sizes compared to the control group. ↑ The survival rate of tumor-carrying mice.	[15]
			Prostate	↑ Inhibition of cell growth inducing apoptosis.	[15]
			Lung	- Inhibited the growth of A-549 cancer cells. ↑ Apoptosis of A-549 cells.	[35]
			Cervical	↓ Cell viability of cancer cells. - Paralyzed the cell cycle in the G2/M phase and cellular apoptosis. - Inhibited cell migration and invasion.	[36]
Curcumin	Non-flavonoid/Phenolic acids	Saffron	Breast	↑ Inhibit the telomerase gene expression in T47D cell line.	[37]
			Cervical	↑ Inhibition of cell proliferation in Hella.	[16]
			Prostate	↑ Uptake of cancer cells DU145. ↑ Therapeutic effect. ↓ Cell viability.	[38]
			Colorectal	- Suppressed tumorigenesis in AOM-DSS in mice. ↓ Decreased both the tumor number and tumor size compared with the AOM-DSS treatment group. ↓ Expression of IL-1β, IL-6, Cox-2, and β-catenin.	[39]
EGCG	Flavonoids/Flavonols	Green tea	Breast	↓ Cellular viability of MCF-7 and MDA-MB-231 strains. ↓ Expression and DNA methyltransferase (DNMT) activity.	[40]
			Prostate	↓ Cell proliferation of DU145. - Induction of apoptosis.	[41]
			Lung	↑ Inhibition of cell proliferation of H1299. - No formation of H1299 colonies.	[42]
Resveratrol	Non-flavonoid/Stilbenes	Grapes, berries, and peanuts	Lung	↓ Cellular viability. ↑ Senescent and apoptotic cells. - Inhibited cell proliferation of A549 and H1299.	[17]
			Cervical	- Inhibited the proliferation and migration of HeLa cells. ↓ Expression of MAPK3.	[31]

Legend: EGCG = epigallocatechin-3-gallate. ↓ = Decrease; ↑ = Increase.

**Table 3:** Some cancer models in preclinical studies from polyphenol delivery systems.

Type of Cancer	Polyphenol	Nanocarrier/ Nanoformulation	Animal Model and Dose/Treatment	In Vivo Effects	Publication Year	References
Breast	EGCG	Self-assembled nanoparticles containing EGCG, Fe^2+^ ions, and DOX	Tumors induced by 4T1 cells in female BALB/c mice using subcutaneous route.	- Remarkable performance in diagnosing tumors using magnetic resonance imaging. - Inhibition of tumor cell metastasis.	2020	[168]
	Lutein	Self-assembled nanoparticles conjugated with DSPE-PEG and folic acid	4T1 cells inoculated into the right breast or tail vein of female BALB/c mice, and administration of luteolin-loaded nanoparticles (10 mg/kg) intravenously.	- Accumulation at tumor sites. - Efficient inhibition of tumor growth regarding free polyphenol. ↓ Systemic toxicity. - Cell death by apoptosis.	2021	[169]
	Podophyllotoxin	PEG polymer micelles modified with T7 peptide and mPEG	MCF-7 cells inoculated subcutaneously into the hind flank of female mice, and intravenous administration of polyphenol-loaded micelles (80 mg/kg).	↑ Maximum tolerated dose of the polyphenolic compound. ↓ Weight loss of animals. - Inhibition of tumor growth.	2019	[170]
	Resveratrol	Folic-acid-linked polymer nanogels	Ehrlich ascites tumor (EAT) cells injected into the mammary gland of female BALB/c mice, and intravenous administration of resveratrol-loaded nanoparticles (2 mg/kg).	- Suppression of tumor growth. ↓ VEGF-1 and Ki-67 expression levels. - Upregulation of caspase-3 (apoptosis induction). - Necrosis in tumor tissues.	2023	[69]
Lung	Catechin	Chitosan-PLGA-based polymer nanoparticles	Nanoparticles administered by different routes (intravenous, oral, intranasal) in Wistar rats.	↑ Bioavailability. - No apparent tissue toxicity regardless of route of administration.	2020	[171]
	EGCG	PLGA nanoparticles	Human lung tumor xenograft implanted in the flank of male NOD/SCID mice and BALB/c mice, and administration intraperitoneally of EGCG-loaded nanoparticles (5 mg/kg).	- No changes in body weight of animals. ↓ Tumor volume and weight. ↓ Expression of Ki-67 protein and negative regulation of phospho-NF-κB. ↑ Cell death by apoptosis.	2020	[66]
	Quercetin	Liposomes modified with RGD peptide	A549 cells injected into the right flank of C57BL/6 mice, and intravenous administration of polyphenol-loaded nanoparticles (5 and 10 mg/kg).	Tumor targeting ability. Considerable half-life and average rate of residence in plasma. ↓ Tumor volume. ↓ Organ toxicity of animals.	2018	[172]
Lung	Resveratrol	Casein nanoparticles	Resveratrol-loaded nanoparticles (15 mg/kg) injected intravenously in Wistar rats.	↑ Availability compared to free polyphenol after oral administration. ↑ Average stay rate and half-life.	2018	[173]
Colorectal	EGCG	Gelatin/chitosan nanoparticles	Mice-bearing orthotopic colon cancer was treated with EGCG-loaded nanoparticles (15 mg/kg) using oral injection.	↑ Half-life improving pharmacokinetics. ↓ Tumor volume. ↑ Tumor inhibition rate. - The appearance of necrosis and apoptosis regions in treated tissue and no damage to non-target organs.	2019	[165]
	Quercetin	PEG-functionalized quercetin nanoparticles	CT26 cells inoculated subcutaneously into the right flank of female BALB/c mice, and polyphenol-loaded nanoparticles (6 mg/kg) inoculated into the caudal vein.	↑ Accumulation at tumor site compared to other organs after 24 h.	2019	[174]
	Resveratrol	PEG-PE polymer micelles	CT26 cells were inoculated subcutaneously in the armpit of female BALB/c mice, and the resveratrol nanoformulation (5 mg/kg) was injected into the animals’ caudal vein.	Longer plasma residence time compared to free polyphenol. ↓ Tumor growth. ↓ Systemic toxicity reflected little change in body weight and lower tumor weight. ↑ Survival over time.	2019	[175]
	Resveratrol	PLGA-PEG nanoparticles coated with chitosan	COLO205-luc cells injected subcutaneously in the axilla of female mice induced a colorectal tumor. An orthotopic model of cancer was treated using resveratrol-loaded nanoparticles (2 mg/kg) introduced into the animals’ cecum.	↓ Tumor growth and angiogenesis. ↑ Bioavailability than polyphenol in free form.	2020	[176]

Legend: EGCG = epigallocatechin-3-gallate; DSPE-PEG = 1,2-distearoyl-sn-glycero-3-phosphoethanolamine-N-methoxy-poly(ethylene glycol); DOX = doxorubicin; PEG = poly(ethylene glycol); PEG-PE = poly(ethylene glycol) phosphatidylethanolamine; mPEG = methoxy-poly(ethylene glycol); PLGA = poly(lactic-co-glycolic acid); RGD = peptide composed of arginine–glycine–aspartic acid. ↓ = Decrease; ↑ = Increase.

## Data Availability

All data will be available upon request.
